# MITF induces escape from innate immunity in melanoma

**DOI:** 10.1186/s13046-021-01916-8

**Published:** 2021-03-31

**Authors:** Luis Sánchez-del-Campo, Román Martí-Díaz, María F. Montenegro, Rebeca González-Guerrero, Trinidad Hernández-Caselles, Enrique Martínez-Barba, Antonio Piñero-Madrona, Juan Cabezas-Herrera, Colin R. Goding, José Neptuno Rodríguez-López

**Affiliations:** 1 Department of Biochemistry and Molecular Biology A, School of Biology, IMIB-University of Murcia, 30100 Murcia, Spain; 2 Department of Biochemistry and Molecular Biology B and Immunology, Faculty of Medicine, IMIB-University of Murcia, Murcia, Spain; 3 Department of Pathology, University Hospital Virgen de la Arrixaca, IMIB, Murcia, Spain; 4 Department of Surgery, University Hospital Virgen de la Arrixaca, IMIB, Murcia, Spain; 5 Translational Cancer Research Group, University Hospital Virgen de la Arrixaca, IMIB, Murcia, Spain; 6 ResearchNuffield Department of Clinical Medicine, Ludwig Institute for Cancer, University of Oxford, Headington, Oxford, OX3 7DQ UK

**Keywords:** MITF, Melanoma, ADAM10, NK-cells, Anti-tumor immunity

## Abstract

**Background:**

The application of immune-based therapies has revolutionized cancer treatment. Yet how the immune system responds to phenotypically heterogeneous populations within tumors is poorly understood. In melanoma, one of the major determinants of phenotypic identity is the lineage survival oncogene MITF that integrates diverse microenvironmental cues to coordinate melanoma survival, senescence bypass, differentiation, proliferation, invasion, metabolism and DNA damage repair. Whether MITF also controls the immune response is unknown.

**Methods:**

By using several mouse melanoma models, we examine the potential role of MITF to modulate the anti-melanoma immune response. ChIP-seq data analysis, ChIP-qPCR, CRISPR-Cas9 genome editing, and luciferase reporter assays were utilized to identify ADAM10 as a direct MITF target gene. Western blotting, confocal microscopy, flow cytometry, and natural killer (NK) cytotoxicity assays were used to determine the underlying mechanisms by which MITF-driven phenotypic plasticity modulates melanoma NK cell-mediated killing.

**Results:**

Here we show that MITF regulates expression of ADAM10, a key sheddase that cleaves the MICA/B family of ligands for NK cells. By controlling melanoma recognition by NK-cells MITF thereby controls the melanoma response to the innate immune system. Consequently, while melanoma MITF^Low^ cells can be effectively suppressed by NK-mediated killing, MITF-expressing cells escape NK cell surveillance.

**Conclusion:**

Our results reveal how modulation of MITF activity can impact the anti-melanoma immune response with implications for the application of anti-melanoma immunotherapies.

**Supplementary Information:**

The online version contains supplementary material available at 10.1186/s13046-021-01916-8.

## Background

Cancer is characterized by deregulated cell proliferation driven by activation of pro-proliferative signaling and senescence bypass [1]. As tumors expand, the probability of metastatic dissemination increases with metastases being the major cause of cancer-related death [2]. Over recent years therapeutic options for many cancers have evolved away from non-specific chemotherapies, toward drugs targeting specific driver pathways and more recently a variety of immunotherapies, many of which target immune checkpoints [3]. Yet despite a significant increase in the effectiveness of anti-cancer therapies, many challenges remain, including understanding how the interaction between cancer cells and the immune system modulate the anti-cancer immune response.

Melanoma, a highly aggressive skin cancer originating from melanocytes, represents an excellent model to examine the bidirectional interaction between cancer cells and the immune system. Cutaneous melanoma exhibits one of the highest mutation burdens of any cancer, especially C > T UV signature mutations [4] reflecting the fact that exposure to solar UV represents one of the primary causes of melanoma initiation. Although early stage melanoma can be effectively cured by surgical excision, metastatic melanoma has historically had a dismal prognosis; once the disease has spread to the main organs, the survival rate can be as low as 7% largely owing to resistance to conventional chemotherapy [5]. Although therapies targeting activated BRAF, a key driver in around 50% of cutaneous melanomas [6], can have a major therapeutic benefit, resistance almost inevitably emerges within a few months [7]. More recently therapies aimed at enhancing the anti-tumor T-cell responses by blocking immune checkpoints or using adoptive T-cell therapy have been employed with considerable success in several tumor types [8], yet a high proportion of patients still fail to exhibit a durable response in melanoma as well as in other cancer types [9].

One of the key determinants of the response to immune checkpoint blockade is the combination of tumor mutation burden together with the degree of intratumor genetic heterogeneity. Evidence suggests that while tumor mutation burden may be important in generating neoantigens, increased genetic heterogeneity may lead to a less favorable outcome with reduced T-cell reactivity [10]. However, although intratumor genetic heterogeneity is important, it may be insufficient to explain how resistance emerges to both immune and targeted therapies [11, 12]. Increasing evidence from a variety of cancer types including melanoma, suggests that plasticity encoded by the cancer genome endows cells with a capacity to switch phenotypes in response to changes in the microenvironment [2, 13, 14], with some phenotypic states able to tolerate exposure to multiple therapeutic modalities. In melanoma, several distinct phenotypic states have been identified [12] with a variety of microenvironmental cues, including hypoxia [15, 16], nutrient limitation [17, 18], BRAF inhibition [19], inflammation and immunotherapy [17, 20], able to induce cells to dedifferentiate and become invasive and therapy-resistant. Understanding how different phenotypes are established and maintained is therefore important if this non-genetic barrier to effective therapy is to be overcome.

A key determinant of melanoma phenotype is the expression and activity of the microphthalmia-associated transcription factor MITF [21]. MITF represents a major coordinator of melanoma cell biology. It promotes survival, differentiation and proliferation, and plays a critical role in regulating melanoma metabolism [22]. Significantly, MITF expression is transcriptionally and translationally suppressed by several microenvironmental signals such that MITF^Low^ cells adopt an invasive, tumor-initiating and drug and immunotherapy-resistant state [12]. Indeed, modulating MITF activity can alter the ability of melanoma cells to respond to therapy [23]. However, whether MITF might control sensitivity of melanoma cells to the immune system remains unclear, and whether modulation of MITF levels using currently available therapeutic modalities might impact the immune response is unknown. This is important since it is possible that different phenotypic subpopulations characterized by distinct MITF levels would respond differently to the innate versus adaptive immune system with major implications for anti-cancer immunotherapies. To address these key issues we developed an immunogenic mouse model, and examined the effect of MITF knockout on the anti-melanoma immune response. The results reveal a critical role for MITF in determining the response to the innate immune system by controlling the shedding of the ligands for natural killer (NK) cells.

## Methods

### Antibodies

Antibodies against the following proteins were used: β-Actin (Merck, Madrid, Spain; monoclonal clone AC-15; #A5441), ADAM10 (Abcam, Cambridge, UK; rabbit polyclonal; #ab1997), N-Cadherin (Merck; rabbit monoclonal; #04–1126), Calreticulin (Thermo-Fisher, Barcelona, Spain; mouse monoclonal; #MA5–11723), CD19-PE-Cy5 (Biolegend, San Diego, CA, USA; clone 6D5; # 115509), CD3ε- FITC (Biolegend; clone 145-2C11; # 100305), CD314 (NKG2D)-PerCP-eFluor 710 (Thermo-Fisher; clone CX5; #46–5882-80), CD335 (NKp46)-APC (Biolegend; clone 29A1.4; #137607), CD45-PerCP-Cy5.5 (Biolegend; clone 30–711; Cat# 103131), CD8a-PE (Thermo-Fisher; clone 53.6.7; # 12–0081-82), F4/80-APC (eBioscience, San Diego, CA, USA; clone BM8; #17–4801-30), FLAG (Merck; monoclonal clone M2; #F1804), HIF1α (Novus, St Charles, MO, USA; monoclonal clone H1alpha67; #NB100–105), MLANA (Abcam, Cambridge, UK; mouse monoclonal; #ab200544), MITF (Millipore; mouse monoclonal; #MAB3747), Rae1δ (Thermo-Fisher; mouse monoclonal; #14–5756-81), HLA-ABC (Thermo-Fisher; mouse monoclonal; #MA5–11723), HLA-E (Thermo-Fisher; mouse monoclonal; #14–9953-80).

### Cell cultures and treatments

HEK293 and melanoma cell lines (IGR37, IGR39, 501mel, A375, SK-MEL-28, G361, and SK-MEL-2) were tested for mycoplasma and authenticated using genotype profiling according to the ATCC guidelines. YAC-1 cells were kindly provided by Dr. Detlef Schuppan (Institute of Translational Immunology and Research Center for Immunotherapy, University Medical Center Mainz, Mainz, Germany) [24]. B16/F10-luc2 mouse melanoma cells were obtained from Caliper Life Sciences (Hopkinton, MA, USA). Cells were cultured in 10% CO_2_ at 37 °C with 1% penicillin–streptomycin EMEM plus 10% fetal bovine serum (FBS). Cell proliferation was evaluated using colorimetric assays to analyze mitochondrial functions (MTT; Merck). For these assays, cells were plated in 96-well plates at a density of 1000–2000 cells/well. For ionizing radiation assays, the cells were irradiated using an Andrex SMART 200E machine (YXLON International, Hamburg, Germany) operating at 200 kV, 4.5 mA with a focus-object distance of 20 cm at room temperature and at a dose rate of 10 Gy. The radiation doses were monitored using a UNIDOS universal dosimeter in a PTW Farme ionization chamber TW 30010 (PTW-Freiburg, Freiburg, Germany) in a radiation cabin. Cobalt chloride (CoCl_2_; Merck) treatment effectively induced the stabilization of HIF1α in melanoma cells. A fresh stock solution 0.4 M CoCl_2_ was prepared in water and added to the medium to obtain desired final concentrations. Cells were incubated with 200 μM CoCl_2_ for indicated periods of time. For glutamine deprived experiments cells were cultured in EMEM without glutamine plus 10% dialyzed FBS.

### CRISPR/Cas9 MITF and ADAM10-knockout

IGR37-MITF-KO, B16/F10-luc2-MITF-KO and B16/F10-luc2-ADAM10-KO cells were generated using the CRISPR/Cas9 technique. Cells were grown up to 60–70% confluency in a 6-well plate and transfected with the Santa Cruz Biotechnology (Dallas, TX, USA) plasmids MITF or ADAM10 CRISPR/Cas9 containing the specifics gRNA sequences and the Cas9 ribonuclease (MITFh #SC-400401-KO-2, MITFm #SC-421654-KO-2, ADAM10m #SC-418981) and their HDR plasmids containing the puromycin resistance gene for selection (MITFh #SC-400401-HDR, MITFm #SC-421654-HDR, ADAM10m #SC-418981-HDR) using the FuGENE6 transfection reagent (Promega, Madison, WI, USA) and a 1:3 DNA/FuGENE6 ratio. The transfection medium was maintained for 48 h. At day 2, medium was changed with fresh medium and puromycin was added at 5 μg/mL. Cells were allowed to grow for three more days. After 5–6 days, single cell colonies were isolated and cell colonies were expanded. Positives clones were characterized by genomic DNA extraction followed by Sanger sequencing and western blot experiments.

### Stealth RNA transfections

Specific Stealth siRNAs for MITF (#HSS142939 and #HSS142940) were obtained from Thermo-Fisher and transfected into melanoma cells using Lipofectamine 2000. Stealth RNA-negative control duplexes (Thermo-Fisher) were used as control oligonucleotides, and the ability of the Stealth RNA oligonucleotides to knockdown the expression of selected genes was analyzed using western blot analysis at 72 h after siRNA transfection.

### PCR analysis

mRNA extraction, cDNA synthesis, and conventional and quantitative real-time RT-PCR were performed under standard conditions [25]. Primers were designed using Primer Express version 2.0 software and synthesized by Thermo Fisher Scientific. The following primers for human genes were used: MITF (forward: 5′-GCG CAA AAG AAC TTG AAA AC-3′; reverse: 5′- CGT GGA TGG AAT AAG GGA AA-3′), ADAM10 (forward: 5′-CTG CCC AGC ATC TGA CCC TAA-3′; reverse: 5′-TTG CCA TCA GAA CTG GCA CAC-3′), HLA-A (forward: 5′-AAA AGG AGG GAG TTA CAC TCA GG-3′; reverse: 5′-GCT GTG AGG GAC ACA TCA GAG-3′), HLA-B (forward: 5′-CTA CCC TGC GGA GAT CA-3′; reverse: 5′-ACA GCC AGG CCA GCA ACA-3′), HLA-C (forward: 5′-ATC GTT GCT GGC CTG GCT GTC CT-3′; reverse: 5′-TCA TCA GAG CCC TGG GCA CTG TT-3′), HLA-E (forward: 5′-CCT ACG ACG GCA AGG A^− 3^′; reverse: 5′-CCC TTC TCC AGG TAT TTG TG-3′), β-actin (forward: 5′-AGA AAA TCT GGC ACC ACA CC-3′; reverse: 5′-GGG GTG TTG AAG GTC TCA AA-3′). Reactions were done in SYBR Green mix (Applied Biosystems, Foster City, CA, USA) using the QuantStudio 5 Real-Time PCR System (Applied Biosystems). Data were analyzed using the 2^-∆∆Ct^ method and relative mRNA expression levels were normalized to β-actin.

### Confocal microscopy

Imaging of fluorescence staining was done by confocal imaging of fixed cells with a laser-scanning confocal inverted microscope (Leica TCS 4D, Wetzlar, Germany), and a 63×/1.4 numerical aperture oil objective was used to image the samples. For indirect immunofluorescence studies, cells were grown on 100-mm^2^ coverslips, fixed in 3% paraformaldehyde and permeabilized (when necessary) with 0.2% Triton X-100. Coverslips were incubated in 5% bovine serum albumin (BSA) for 20 min and probed with primary antibodies (diluted 1:200 in PBS containing 5% BSA) for 2 h at room temperature. Cells were washed three times in PBS and incubated for 1 h at room temperature with Alexa Fluor Dyes [Alexa Fluor 488 goat anti-mouse IgG (H + L) (#A11001) and Alexa Fluor 633 goat anti-rabbit IgG (H + L) (#A21071) both from Thermo-Fisher]. Coverslips were permanently mounted to the slides using fluorescent mounting medium (PROLONG-GOLD, Thermo Fisher Scientific).

### Western blots

Whole-cell lysates were collected by adding SDS-PAGE sample loading buffer. After sonication, the samples were boiled (10 min) and proteins were separated by SDS-PAGE, transferred to nitrocellulose membranes and analyzed using immunoblotting (WesternBright Quantum, Advansta, San Jose, CA, USA).

### Flow Cytometry

Samples were analyzed using flow cytometry in a FACSort cytometer (BD, Franklin Lakes, NJ, USA) and Cell Quest (BD) and by FlowJo software version 10.2 (FlowJo, Ashland, OR, USA) [26]. Analysis of the immunoinfiltrate in mice lungs was performed by Flow cytometry of single cell suspensions. For this, lungs were collected, washed in PBS, minced and digested using Collagenase IV (Merck) at 37 °C for 30 min. After incubation, the remaining tissues were filtered through a 70 μm filter (BD) and washed in complete EMEM medium. After isolation by Ficoll density gradient, leukocyte were stained for flow cytometry analysis. Briefly, 0.3 × 10^6^ cells were incubated with anti-CD3-FITC, anti-NKp46-APC and anti-CD45-PerCP-Cy5.5 antibodies for 15 min in the dark, fixed (1x BD lysis solution), washed (PBS) and then were acquired in a LSR Fortessa X-20 Cytometer (BD). To analyse data, lymphocyte region was selected on the forward scatter versus side scatter plot and then gated on the CD45+ cells to exclude cell debris and non-hematopoietic cells. NK cells were identified as CD3−/NKp46+ cells.

### ChIP assays

The ChIP assays were performed with the Magna ChIP kit (#17–10,085) from Merck according to the manufacturer’s instructions. Briefly, 501mel or IGR37 melanoma cells were cross-linked with formaldehyde 0.4% and the reaction was stopped by adding glycine to final concentration 0.2 M. Cells where lysated and DNA was sheared by sonication to give an average size of 300 to 3000 bp. The cross-linked chromatin was then used for immunoprecipitation with MITF antibody, HDAC3 antibody (positive control) or mouse IgG (negative control). DNA from lysates prior to immunoprecipitation was used as positive input controls. After washing, elution, and DNA purification, the DNA solution (2 μl) was used as a template for real-time PCR amplification using specific human primers: ADAM10 (forward: 5′-GCG CGT CAC GTG GTG AGG AA-3′; reverse: 5′-CCC TGG CAG GAG AAA CGG CG-3′); and GAPDH (forward: 5′-CAA TTC CCC ATC TCA GTC GT-3′; reverse: 5′-TAG TAG CCG GGC CCT ACT TT-3′). Negative control region for SILV (forward: 5′-CAT GGA GAA CTT CCA AAA GGT GG-3′; reverse: 5′-TAC TCT CCC CAG GGA GTA TAA GT-3′) were also used for PCR amplification. Standard curves were generated for all primer set to confirm linearity of signals over the experimentally measured ranges.

### Luciferase assay

ADAM10 LightSwitch Promoter Reporter GoClones (RenSP, #S722690) and empty promoter vectors (#S790005) were obtained from SwitchGear Genomics (Menlo Park, CA, USA) and transfected into HEK293 cells using FuGENE6 according to the manufacturer’s instructions. MITF-FLAG was made by cloning the MITF-M cDNA into p3xFLAG-CMV-14 (Merck). Cells were lysated 48 h after transfections and luciferase activity was measured in triplicates using LightSwitch luciferase Assay Reagent (SwitchGear Genomics) and Fluostar Omega plate reader (BMG Labtech., Ortenberg, Germany). The mutant for the ADAM10 promoter reporter was made using its LightSwitch Promoter Reporter as template, changing CACGTG to CTGGTG in the MITF binding sequence (E-Box). Point mutations were created using the QuikChange Lightning Site-Directed Mutagenesis kit (Agilent Technologies, Santa Clara, CA, USA) following the manufacturer’s recommendations together with the following mutagenesis primers: M-F, 5′-GCC CGC GCG TCA CCA GGT GAG GAA GGA G-3′ and M-R, 5′-CTC CTT CCT CAC CTG GTG ACG CGC GGG C-3′.

### Isolation of NK cells and NK cytotoxicity assays

Murine NK cells were isolated from spleens of C57BL/6 or Nude-Foxn1nu mice. Spleens were mechanically dissociated, and mononuclear cells were isolated by Ficoll density gradient centrifugation. Cells were washed with PBS and NK cells were enriched by negative selection using the Mouse NK Cell Isolation Kit II (#130–096-892, Miltenyi Biotech, Auburn, CA, USA). Cells were then incubated with anti-NKp46-APC, anti-CD3-FITC and -anti-CD19-PE-Cy5 to confirm the correct NK cell purification. When mice were no previously immunized, NK cells were activated with 200 units/ml IL-2 (Thermo-Fisher). Target cells were labeled for 1.5 h with 100 μCi of ^51^Cr/10^6^ cells in 0.2 ml of media in a 5% CO_2_, 95% humidified atmosphere incubator at 37 °C. To determine the experimental release of ^51^Cr (Perkin Elmer, Waltham, Massachusetts, USA), NK cells were added in an equal volume, but at different ratios. To determine maximum and spontaneous release of ^51^Cr, target cells were mixed with an equal volume of 2% Triton-X100 in PBS or an equal volume of media, respectively. Plates were then spun for 1 min at 1000 rpm. NK cells and target cells were incubated for 4 h at 37 °C 5% CO_2_. After incubation, plates were centrifuged (2000 rpm, 5 min) and 100 μl of supernatant was collected, mixed with scintillation fluid, and radioactivity was quantified using a MicroBeta2 reader (Perkin Elmer). Specific killing (^51^Cr release) was calculated using the following formula: 100 x (Experimental release - Spontaneous release)/(Maximum release - Spontaneous release).

### ELISA assays

Soluble forms of NKG2D ligands MICA and MICB were quantified in cell free supernatants using sandwich ELISA following the manufacturer’s instruction (Human MICA #RAB0358 from Merck and Human MICB #BMS2303 from Thermo-Fisher). Absorbance was measured at 450 nm with a reference wavelength of 620 nm in a Bio-Rad Filter-based microplate spectrophotometer (Bio-Rad Laboratories, Hercules, CA, USA). ELISA absorbance values were proportional to the concentration of protein stablished with lyophilized standards. Apoptosis was quantified using a Cell Death Detection ELISA^PLUS^ kit (Roche Diagnostics, Barcelona, Spain) following manufacture’s protocol.

### Mice

Female C57BL/6, Rag2−/− Il2rg−/−, Athymic Nude-Foxn1nu mice, 4–5 weeks of age, were all obtained from Envigo (Barcelona, Spain). Mice were housed under aseptic conditions (positive air pressure in a designated mouse room with microisolator tops), in the specific pathogen free animal facility at University of Murcia and all mouse handling procedures were carried out under a laminar flow hood. Primary tumors were stablished subcutaneously with 5 × 10^5^ B16/F10 cells with luciferase expression. For metastases colonization experiments, 5 × 10^5^ B16/F10 cells expressing luciferase were injected intravenously into mice through the tail vein. Primary tumors and metastases were analyzed using the IVIS Imaging System (Caliper Life Sciences, Hopkinton, MA, USA). For CD8+ T cell depletion experiments, 250 μg anti-CD8 (Bio-XCell, West Lebanon, NH, USA; clone 2.43; #BE0117) per mouse was delivered four times by i.p. injection every 3 days. We made every attempt to reach the conclusion using as small sample size as possible. We usually exclude samples if we observe any abnormality in terms of size, weight or apparent disease symptoms in mice before performing experiments. However, we did not exclude any animals here, as we did not observe any abnormalities in the present study. Neither randomization nor blinding was performed in this study.

### Histology and immunohistochemistry

Mice were sacrified 3 weeks after tail vein injections when the presence or absence of metastases was assayed using the IVIS imaging System. Lungs were removed, washed in PBS and fixed in 10% formalin. Routine hematoxylin and eosin (H&E) staining was performed on serial sections of paraffin embedded tissues. Light microscope photographs of the stained sections were collected using a Nikon Eclipse 90i microscope and images were analyzed using ImageJ software. For immunohistochemistry, paraffin sections were deparaffinized and subjected to antigen retrieval in sodium citrate buffer (pH 6.0). Tissue endogenous peroxidase was inactivated with 5% hydrogen peroxide. Slides were blocked and incubated with a NKp46 primary antibody (#PA5–79720, Thermo-Fisher) overnight at 4 °C. Next, slides were incubated with secoundary antibodies conjugated with peroxidase and specific reaction was developed with 0.2% diaminobenzidine tetrahydrochloride and hydrogen peroxide.

### Quantification and statistical analysis

Western blot analyses and analyses of microscopy data were performed as we have previously described [26]. Analyses were repeated at least three times, with similar results. The results from one experiment are shown. To quantify the results, the western blots were scanned using a Bio-Rad ChemiDoc scanning densitometer (Bio-Rad Laboratories). Fluorescence intensity versus cell number analysis in confocal microscopy experiments was performed with ImageJ-NIH (Bethesda, MD, USA). For other experiments, the mean ± S. D of three measurements performed in triplicate were calculated. Numerical data were analyzed to determine statistical significance using Mann–Whitney tests for comparisons of means in the SPPS statistical software for Microsoft Windows, release 6.0 (Professional Statistic, Chicago, IL, USA). Individual comparisons were made using Student’s two-tailed, unpaired t-tests. The criterion for significance was *p* < 0.05 for all comparisons. Normality was tested using the Shapiro-Wilk test.

## Results

### Ex vivo irradiation induces immune suppression of melanoma tumor formation

**Fig. 1:**
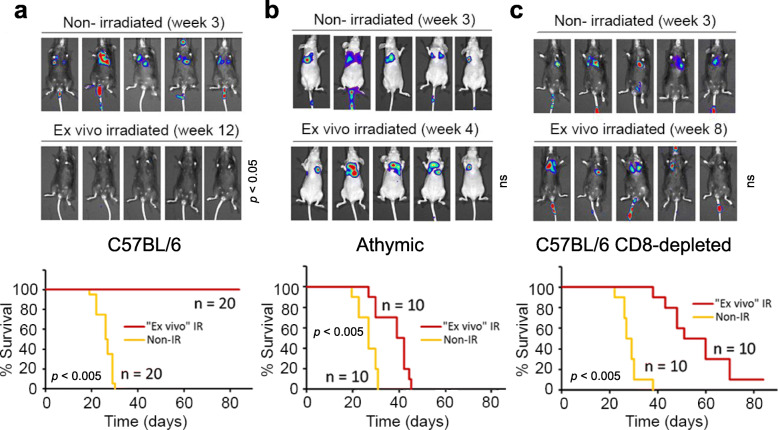
The effect of ex vivo irradiation on the immune response against melanoma. **a to c** Luciferase imaging of C57BL/6 **a, c** and athymic **b** mice injected in the tail vein with B16/F10 cells. When indicated, cells were irradiated ex vivo (10 Gy; 24 h) or mice were treated with an anti-CD8 antibody **c**. Total flux (photons/s) analyzes were compared between ex vivo irradiated and non-irradiated groups (ns, no significant). Survival in the melanoma mouse models was examined (Graphs below).*p*-values indicate significant differences in survival when compared both tested groups. Color scale was set to Min = 1.00e6; Max = 1.00e7 for all pictures

One of the defining features of human melanoma is its unusually high mutation burden that is thought to increase its potential recognition by the immune system. However, mouse melanomas in general are poorly immunogenic. For example, the widely used B16/F10 melanoma cell line has been defined as poorly immunogenic based on its ability to form consistent subcutaneous tumors in syngeneic C57BL/6 mice [27]. However, irradiation of mouse melanoma cell lines can generate neoantigens that improve immune recognition and recapitulate the genetic heterogeneity associated with the human disease [10]. As a first step in assessing the potential impact of MITF on the anti-melanoma immune response, we developed an immunogenic B16/F10 melanoma model that accurately reflects the human disease by subjecting cells to irradiation prior to inoculation into immunocompetent mice. To this end, we injected cells into the tail vein of syngeneic C57BL/6 mice either naïve B16/F10 luciferase-expressing melanoma cells or those exposed to 10 Gy 24 h prior to injection, and assessed their ability to initiate colonies within the lung. The results were striking. In immunecompetent C57BL/6 mice, non-irradiated cells readily colonized the lungs, the initial destination of tail vein injected cells (Fig. 1a). By contrast, using irradiated B16/F10 cells, we observed a complete reduction of tumor load. Compared with animals injected with untreated B16/F10 cells, mice injected with irradiated cells were free of disease and with a longer and indefinite time of survival. To determine whether this effect was mediated by the immune system and not due to reduced viability and/or colonization capacity of the irradiated cells, cells were also injected into an athymic nude mouse model (Hsd: Athymic Nude-Foxn1nu), lacking T cells but maintaining the functionality of B and NK cells. In this model the effectiveness of cell irradiation in suppressing lung colonization was severely reduced, resulting in rapid tumor formation (Fig. 1b). In agreement with this observation, inactivation of CD8+ T cells using a specific antibody (Fig. S1) abolished some of the effect of irradiated cells in immunecompetent mice, resulting in tumor growth but with a delayed formation that resulted in prolonged survival (Fig. 1c). These observations indicate that irradiated melanoma cells elicit an anti-melanoma immune response mediated in part by the adaptive immune system.

To confirm that irradiation modulated the immune responsiveness of melanoma cells, we next examined the effect of irradiation on the expression of MHC class I genes that are required for antigen presentation and immune recognition. Using an anti-HLA-ABC antibody, we observed that IR increased the expression of these MHC class I molecules as detected by immunofluorescence (Fig. S2a) that was reflected in elevated cell surface expression detected by flow cytometry (Fig. S2b) of both HLA-ABC as well as HLA-E. Using qRT-PCR to determine the mRNA levels of these four MHC class I molecules in melanoma cells after IR, demonstrated a significant increase of HLA-A and-E mRNA, but not for HLA-B and -C mRNA (Fig. S2c). We also noted a substantial increase in cell surface expression of calreticulin (Fig. S2d) in human SK-MEL-28 melanoma cells, and especially in the IGR39 cell line that is known to be BRAF inhibitor tolerant and displays gene expression signatures linked to immunotherapy resistance [28]. Since cell-surface expression of calreticulin is important for recognition by dendritic cells [29], the increased expression of calreticulin would also be consistent with an elevated anti-melanoma cell immune response to irradiated cells.

### Ionizing irradiation activates MITF

Our results indicate that irradiation of B16/F10 melanoma cells increases their immunogenicity, likely in part via increased MHC class-I mediated antigen presentation, raised calreticulin expression to promote recognition by dendritic cells, and elevated expression of melanoma-associated antigens such as MLANA (Fig. S2d). The melanoma-associated antigen recognized by T-cells (MLANA) encoded by the *MLANA* gene is one of the major melanoma tumor antigens linked to immune recognition [30]. Since expression of MLANA, a differentiation-associated melanosomal protein, is regulated by MITF [31], our results suggested that irradiation might also induce MITF expression, and that MITF could play a role in immune recognition of melanoma cells.

**Fig. 2:**
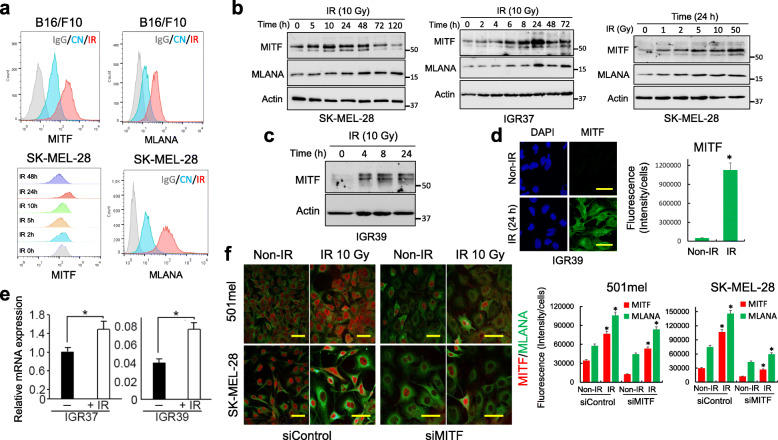
Effect of IR on MITF expression. **a** Flow cytometry analysis of MITF and MLANA in different melanoma cell lines and effect of IR (10 Gy, 24 h). Quantitative analysis is showed in Fig. S7. **b** Time and dosage effect of IR on the expression of MITF and MLANA analyzed by Western blot. In all cases, β-actin was used as a load control. **c** Effect of IR on MITF expression in IGR39 melanoma cells analyzed by Western blot. **d** Confocal microscopy analysis of MITF in IGR39 melanoma cells under indicated conditions (IR, 10 Gy). (Bars, 15 μm). *p < 0.05 when compared fluorescence in IR vesrsus non-IR groups. **e** qRT-PCR analysis of MITF mRNA in indicated melanoma cells before and after IR (10 Gy; 24 h). Relative mRNA expression in IGR39 cells was normalized with respect to untreated IGR37 cells. *p < 0.05. **f** Confocal microscopy analysis of MITF and MLANA before and after IR (24 h) in siControl and siMITF transfected melanoma cells. (Bars, 15 μm). Histograms represent the fluorescent intensity per cell in different assayed conditions. *p < 0.05 when compared fluorescence in IR versus non-IR groups. Quantification of blots presented in this figure can be visualized in Fig. S8. The groupings blots in this figure were cropped from different gels. Full blots are shown in the Supplementary Information, Fig. S9

To investigate this possibility, we undertook flow cytometry analysis of the B16/F10 melanoma cells used for the tumor formation assays, using antibodies specific for MITF and MLANA. The results (Fig. 2a, top panels) revealed that irradiation increased expression of both proteins, a result also reflected in the radiation-induced increased expression of MITF and MLANA in human SK-MEL-28 melanoma cells (Fig. 2a, lower panels). Western blotting in both SK-MEL-28 and IGR37 cells confirmed the transient nature of the irradiation-dependent induction of MITF (Fig. 2b), with MLANA expression increasing after that of MITF, consistent with it being an MITF target gene. The effects of radiation were also dose dependent (Fig. 2b, right panel). In addition to the MITF^High^ (IGR37, SK-MEL-28) cell lines we also used the MITF^Low^ mesenchymal phenotype melanoma IGR39 cell line. Remarkably, although this cell line expresses extremely low levels of MITF, irradiation induced robust MITF protein expression within 4 h as detected by western blotting (Fig. 2c) or immunofluorescence (Fig. 2d). The changes in MITF protein levels in IGR37 and IGR39 cells were reflected in a moderate increase in mRNA following irradiation (Fig. 2e). The induction of MLANA was confirmed to be dependent on MITF, since depletion of MITF using siRNA prevented the irradiation-dependent increase in MLANA expression in human melanoma cell lines (Fig. 2f). Collectively these observations indicate that MITF can be induced in response to irradiation, with increased MLANA antigen expression correlating with the irradiation-induced immune response that prevented tumor formation in mice.

### MITF-depleted melanoma cells are susceptible to a T cell-independent immune response

To examine the potential role of MITF in the immune response to melanoma, we generated MITF knockout cell lines (B16/F10-MITF-KO and IGR37-MITF-KO) using CRISPR-Cas9. The mutated sequences are shown in Fig. S3a and Fig. S3b, and lack of MITF expression was detected by Western blot in Fig. S3c. Knockout of MITF in either IGR37 or B16/F10 cells led to an altered cell morphology (Fig. S3d), moderately reduced proliferation (Fig. S3e, left panels) but no increased apoptosis, with doxorubicin treatment used as a positive control (Fig. S3e, right panels). As expected, the B16/F10-MITF-KO cell line failed to induce MITF following irradiation (Fig. S3f). Since MITF has been associated with regulation of pro-proliferative and pro-survival genes [22], we verified that the B16/F10-MITF-KO cells (clone 3–1) were able to form tumors in different experimental models. First, we observed that both parental and B16/F10-MITF-KO cells were consistently able to induce lung tumors in tail vein injected Rag2/IL2RG mice (R2G2), a double knockout mouse with an ultra immunodeficient phenotype, independently of their irradiation status (Fig. S4a). In addition, like the parental cell line, B16/F10-MITF-KO cells consistently formed tumors when they were injected subcutaneously (Fig. S4b). To rule out any potential immunogenicity of the product of the puromycin resistance gene used to select for CRISPR MITF-KO cells or luciferase expressed protein, B16/F10-luc2 parental cells were transfected with the same puromycin selection plasmid as the KO cells. After selection, irradiated cells were injected into the tail vein of athymic nude mice. As expected lung metastases developed similar to those injected with irradiated parental cells (Fig. S4c).

**Fig. 3:**
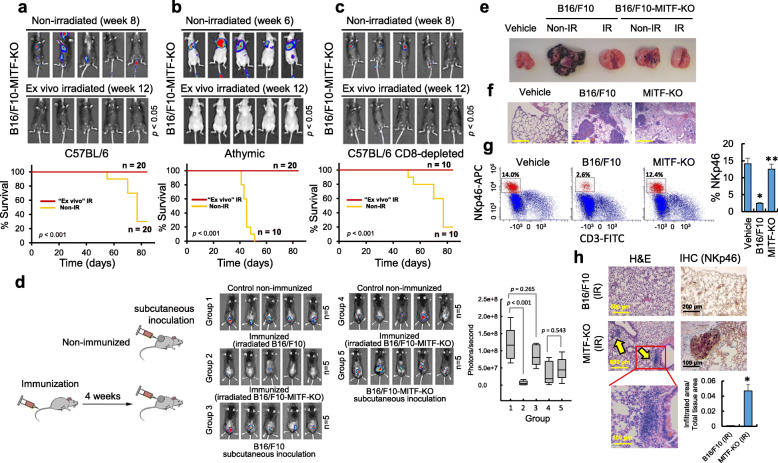
Effect of MITF on the anti-melanoma immune response. **a to c** Luciferase imaging of C57BL/6 **a, c** and athymic **b** mice after tail vein injection with B16/F10-MITF-KO cells. The experimental conditions were as those described in Fig. 1. **d** Vaccination assay. Tumors in non-immunized animals subcutaneously injected with irradiated B16/F10 and B16/F10-MITF-KO cells (Groups 1 and 4, respectively). Animals in Group 2 were immunized with ex vivo irradiated B16/F10 cells. After 4 weeks, non-irradiated B16/F10 cells were injected subdermally. Groups 3 and 5 were immunized with ex vivo irradiated B16/F10-MITF-KO cells. After 4 weeks, non-irradiated B16/F10 (Group 3) or non-irradiated B16/F10-MITF-KO (Group 5) cells were injected subdermally. Analysis of primary tumors was carried out 4 weeks after subcutaneous injection. Color scale for Groups 1–3 (Min = 1.00e6; Max = 1.00e7) and for Groups 4–5 (Min = 5.00e4; Max = 5.00e5). **e** Lungs from mice injected in the tail vein with vehicle (PBS) or indicated cells. **f** Microscopic view of the lung tissue structure (H&E stain, × 100) (Bars, 600 μm). **g** Analysis and quantification (histogram) of NKp46-positive cells in mouse lungs performed by flow cytometry of single cell suspensions. *p < 0.005 when compared with vehicle-treated group. **p < 0.005 when compared with B16/F10-treated group. **h** H&E stain and IHC (with anti-NKp46) of lung tissues. Yellow arrows indicate areas of immune infiltration. The proportion of infiltrated area per total area of lung H&E slide (× 40) was calculated by using ImageJ software (histogram). Quantification was carried out on three different areas of each lung and in 3 animals per group (*p = 0.0026). In panels **e to h**, lungs were obtained 3 weeks after vehicle/cells injection and *n* = 3 per group

However, and despite the ability of B16/F10-MITF-KO cells to form tumors in vivo, when these cells were injected into the tail vein of immunecompetent C57BL/6 mice, we observed a similar response to that seen after the injection of parental cells (Fig. 3a), namely that irradiated cells failed to colonize the lungs. By contrast, a remarkably different response was observed after injection of irradiated B16/F10-MITF-KO cells into athymic nude mice (Fig. 3b) where non-irradiated cells readily formed lung tumors, but no tumor formation was observed after injection of irradiated MITF-KO cells. Since parental cells expressing MITF readily form tumors in the athymic mice (Fig. 1b), these results indicate that the response of animals to irradiated B16/F10-MITF-KO cells was T-cell-independent. To confirm this hypothesis we carried out two independent experimental strategies. First, we depleted CD8+ T cells activity using specific antibodies in animals injected with B16/F10-MITF-KO cells (the knockdown effect of the CD8 antibody can be visualized in Fig. S1). As expected, and in contrast to the MITF-positive parental cells (Fig. 1c), inactivation of CD8+ T cells did not increase tumor load in mice injected with irradiated B16/F10-MITF-KO cells (Fig. 3c). We next employed a vaccination assay where animals were immunized 4 weeks before by tail vein injection of irradiated B16/F10 cells. In this case, immunization demonstrated a substantial protective immune response against the subcutaneously inoculated non-irradiated B16/F10 cells (Fig. 3d; compare Group 1 to Group 2). By contrast, protection was substantially reduced if irradiated B16/F10-MITF-KO cells were used for prior immunization (Fig. 3d; compare Group 3 to Group 2). Immunization with irradiated B16/F10-MITF-KO cells also failed to protect against tumor growth following subcutaneous inoculation of B16/F10-MITF-KO cells (Fig. 3d; compare Groups 4 and 5). The immunization experiments indicate that loss of MITF reduces the ability of B16/F10 cells to induce immunological memory and to be targeted by an immune response elicited by parental B16/F10 cells.

### MITF modulates the response of the immune system toward melanoma cells

To better understand the differential response of mice to melanoma injected cells, we next carried out macroscopic and microscopic analysis of the lungs from mouse injected with non-irradiated or ex vivo irradiated cells. As expected, the lungs of mice injected with non-irradiated cells (B16/F10 or B16/F10-MITF-KO cells) showed consistent melanoma tumors (Fig. 3e and Fig. 3f). Although infiltration of immune cells in these H&E preparations was not evident, a cytometry study to detect NKp46-positive cells in extracts of lungs demonstrated that the NK population was diminished in the lungs of mice injected with B16/F10 parental cells when compared with the NK population in the lungs of the vehicle- or B16/F10-MITF-KO-treated mice (Fig. 3g). It is well known that NK cells become progressively exhausted in the context of melanoma progression [32], and these data would be consistent with expression of MITF in melanoma cells influencing the exhaustion of these innate immune cells. Whether the maintenance of this NK population in lungs of B16/F10-MITF-KO-treated mice is responsible for a slower colonization of the lungs when compared to the parental cells (Fig. 3e) or is more related to MITF’s pro-proliferative role are aspects that remain to be investigated.

On the other hand, and consistent with the lack of macroscopic evidence of tumor colonization in the lungs of mice injected with ex vivo irradiated B16/F10 cells (Fig. 3e), histologic analysis indicated that these lungs showed evident signs neither of microlesions nor increased inflammatory infiltrates when compared when the lungs of vehicle-treated controls (Fig. 3f and Fig. 3h). However, the lungs of mice injected with ex vivo irradiated B16/F10-MITF-KO cells, although free of melanoma lesions (Fig. 3e), showed evident areas infiltrated with immune cells (Fig. 3h). Immunohistochemical study with NKp46 antibody was performed in both cell-irradiated groups. Positive NKp46 cells were evident in the inflammatory foci found in the B16/F10-MITF-KO irradiated group whereas the sample with irradiated B16/F10 did not show NK positivity in the lung (Fig. 3h). Collectively, the results indicate that the presence of MITF could differentially modulate the response of the immune system toward irradiated melanoma cells. Thus, although the T-cell-mediated immune response responsible for the elimination of the irradiated B16/F10 cells may prevent the colonization of the lung parenchyma, the presence of NK-positive immune infiltrates in the lung parenchyma of mice injected with B16/F10-MITF-KO might be better explained by immunological activation of NK cells.

### MITF promotes NKG2D ligand shedding in melanoma cells

The results indicate that MITF shapes the anti-tumor immune response; MITF-KO cells are targeted preferentially by the innate immune response, whereas MITF-expressing cells are primarily targeted by T-cells. To investigate the underlying mechanism, we examined the ability of MITF-KO vs MITF parental cells to be engaged by natural killer (NK) cells that represent critical components of the innate immune response and in particular the ability of MITF to regulate melanoma cell surface expression of the MICA and MICB NK-cell ligands. MICA and MICB (Rae1 in mouse) have a key role in the recognition of tumor cells by the innate immune system [33] where their engagement with NKG2D receptors on NK cells triggers NK cell-mediated cytotoxicity. However, advanced cancers frequently escape this immune mechanism by proteolytic shedding of cell surface-bound MICA and MICB molecules through the coordinate action of ERp5 and several cell surface proteases [34]. Consistent with this, high serum concentrations of shed MICA are associated with disease progression in many human cancers, including melanoma [35], where vemurafenib and histone deacetylase inhibitors (such as sodium butyrate) differentially modulate expression of NKG2D ligands such as MICA [36].

**Fig. 4:**
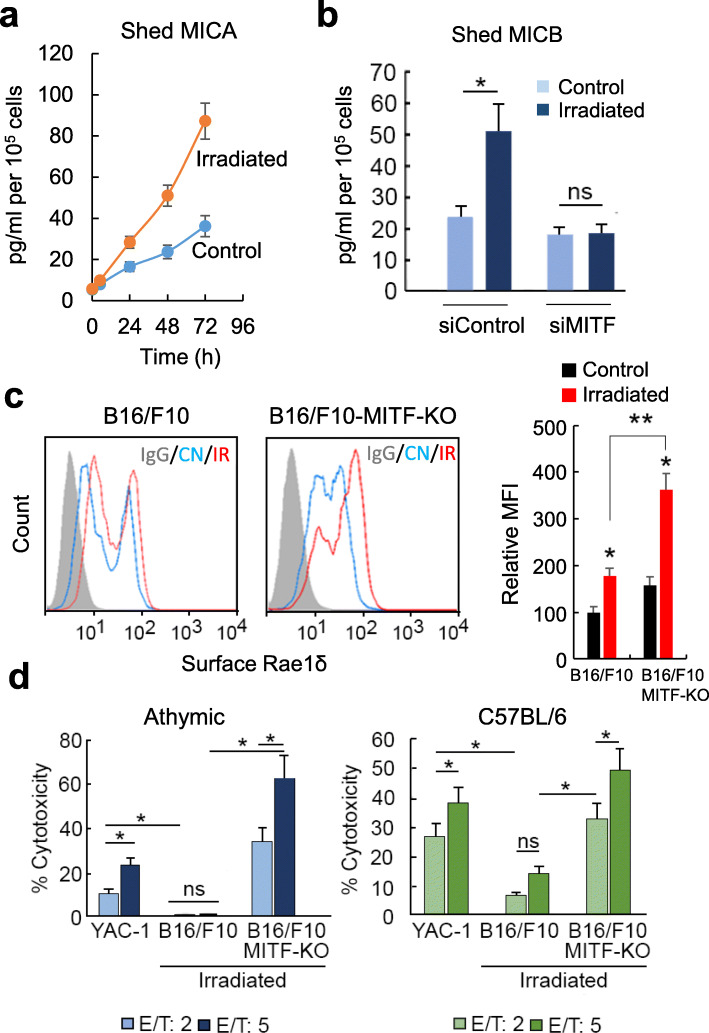
MITF promotes NKG2D ligand shedding in melanoma cells. **a** ELISA assay in SK-MEL-28 cells to determine the concentration of MICA in the extracellular medium. The data show the mean ± SD and the variation in the concentration of MICA after irradiation (IR, 10 Gy) was statistically significant with respect to the control at all times tested (*p <* 0.05). **b** ELISA assay in SK-MEL-28 cells to determine the concentration of MICB in the extracellular medium (*p < 0.05; ns, no significant) MICB was determined at the same time (24 h) in control and irradiated (IR, 10 Gy) cells. **c** Flow cytometry determination of Rae1δ in B16/F10 and effects of MITF depletion. Cells were non-irradiated or irradiated (IR, 10 Gy, 24 h). Histogram represents MFI (Mean Fluorescence Intensity) in assayed conditions. *p < 0.05 when compared with their respective non-irradiated cells. **p < 0.005 when comparing both irradiated cells. **d** Athymic (Fox1nu) and C57BL/6 mice were immunized by injection in the tail vein with ex vivo irradiated B16/F10 cells (10 Gy; 24 h). One week after the injection, animal were sacrificed and NK cells were purified from their spleens. Purified NKs were co-cultured with YAC-1 cells, irradiated B16/F10 or irradiated B16/F10-MITF-KO. NK cytotoxicity was evaluated by a ^51^Cr-release assay at two effector/target ratios (E/T). The results are representative of three independent experiments (*p < 0.05)

Given the critical role of MICA and MICB in the innate immune response to melanoma, we initially used an ELISA assay to examine the amount of MICA in the medium of irradiated cells compared to the non-irradiated control. The results (Fig. 4a), revealed that irradiation induced a substantial increase in shed MICA over time. Significantly, siRNA-mediated depletion of MITF blocked the radiation-induced increase in extracellular MICB (Fig. 4b), consistent with MITF knockdown preventing the shedding of NKG2D ligands in melanoma. To confirm the effect of MITF on NKG2D ligands, we analyzed by flow cytometry cell surface expression of Rae1δ, one of several known murine NKG2D ligands in parental B16/F10 cells or those lacking MITF. The results (Fig. 4c), revealed that when compared with the irradiated parental cell line, knockout of MITF in B16/F10 cells resulted in increased basal levels of Rae1δ as well as in greater accumulation of membrane-bound Rae1δ after IR.

Since MITF induced the proteolytic cleavage of NKG2D ligands, we next examined the activity of NK cells against melanoma cells lacking MITF. To this end, irradiated B16/F10 cells were injected into the tail vein of either C57BL/6 or athymic nude mice. After 1 week, NK cells from these mice were purified from their spleens and co-cultured at two different effector/target ratios (E/T) with irradiated B16/F10, or B16/F10-MITF-KO cells, as well as with the moloney leukemia virus-transformed mouse lymphoma cell line YAC-1 used as a control [24]. Cytotoxicity was determined using a ^51^Cr-release assay. The results (Fig 4d) revealed that NK cells obtained from both C57BL/6 and athymic nude mice were significantly more active toward YAC-1 and B16/F10-MITF-KO cells, while the cytotoxicity of NK cells on B16/F10 parental cells was rather low.

### ADAM10 is a direct MITF target gene

Our results indicate that loss of MITF reduces the shedding of the NKG2D ligands and enhances NK cell-mediated killing of melanoma cells. Key to the cleavage of the MICA and MICB NKG2D ligands is the ADAM (a disintegrin and metalloproteinase) family of metalloproteases, membrane anchored enzymes that are involved in various biological events, such as cell adhesion, cell fusion, cell migration, membrane protein cleavage and proteolysis [37, 38]. Indeed, some ADAMs are expressed in malignant tumors and participate in cancer pathology with ADAM10 and ADAM17 playing a prominent role for this mechanism of tumor immune-evasion by controlling MICA and MICB [39]. Notably, a high percentage of melanoma cells are positive for ADAM10, suggesting that it may regulate the immune response during the onset and progression of melanoma [39].

**Fig. 5:**
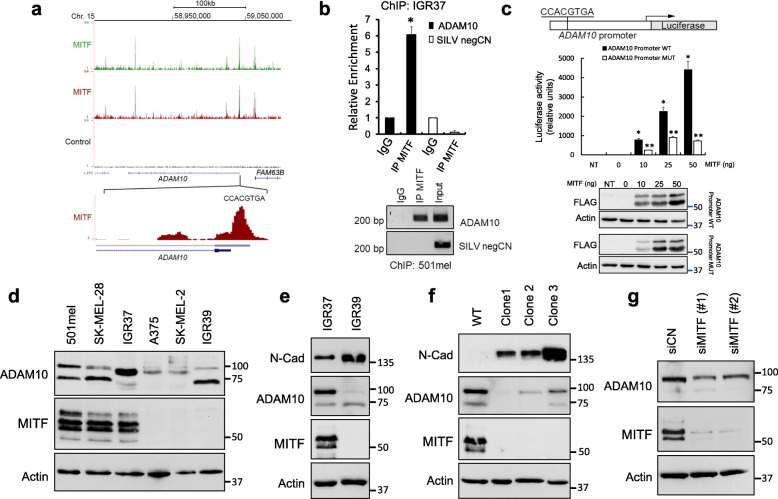
MITF binds to the ADAM10 promoter. **a** UCSC genome browser screenshot showing a biological replicate ChIP-seq of MITF bound to the *ADAM10* locus [40]. **b** Histogram represents ChIP assays in IG37 cells (*p < 0.05). Agarose gels using the PCR products of the ChIP experiments on 501mel cell lines are presented. **c** HEK293 cells were transfected either with a plasmid containing the promoter sequence of ADAM10 upstream of the luciferase-coding sequence (Promoter WT) or with a plasmid contained an ADAM10-mutated promotor sequence (Promoter MUT) in the absence or the presence of overexpressed MITF. The analysis of MITF expression was carried out by western blot assays by using an anti-FLAG antibody, while actin protein was used as load control. *p < 0.05 when compared with MITF negative cells. **p < 0.05 when compared with promoter WT. NT, negative transfected cells. **d** Western blots of different melanoma cell lines, both MITF positive (501mel, SK-MEL-28 and IGR37) and MITF negative (A375, SK-MEL-2, and IGR39) to analyze the levels of MITF and ADAM10 proteins. **e** Western blots to analyze N-Cad, MITF, and ADAM10 proteins in IGR37 cells (MITF positive) and IGR39 cells (MITF negative). **f** Western blots to analyze N-Cad, MITF, and ADAM10 proteins in IGR37 cells and several MITF-KO clones. **g** Effect of MITF silencing on the expression of N-Cad, MITF, and ADAM10 in SK-MEL-28 cells. Blots in this figure were cropped from different gels. Full blots are shown in Fig. S9 and quantified in Fig. S8

Interestingly, both the mouse and human ADAM10 promoter, but not that of ADAM17, contain an E-Box consensus sequence (CCACGTGA) that matches the core MITF binding sequence including a 3′ A residue that enhances MITF binding (Fig. 5a). Examination of a duplicate MITF ChIP-seq dataset [40] revealed that ADAM10 is bound both within the promoter as well as in a 3′ intron (Fig. 5a), although only the promoter binding site contained the MITF consensus sequence. Binding to the intronic sequence may indicate indirect binding by MITF, for example by gene looping. Similar binding by MITF to the same site in the *ADAM10* gene was also confirmed in an independent ChIP-seq dataset [41] (Fig. S5a). To validate the ChIP-seq data, we performed chromatin immunoprecipitation experiments on the MITF^High^ IGR37 and 501mel melanoma lines using an anti-MITF antibody, and then performed PCR assays using primers specific to the *ADAM10* promoter region spanning the binding site identified in the ChIP-seq experiments. As shown in Fig. 5b, specific enrichment of the *ADAM10* promoter region was detected using the anti-MITF antibody that was not observed using an IgG control. As a second negative control, no amplification was observed after anti-MITF chromatin immunoprecipitation across an intron of the *SILV* gene where MITF has no binding capacity. Using an *ADAM10* promoter-luciferase reporter we confirmed that transfecting increasing amounts of an MITF expression vector led to increased promoter activity (Fig. 5c). These data indicate that MITF directly binds and regulates *ADAM10* gene expression.

The observation that MITF controls ADAM10 expression highlights the importance of melanoma phenotype on recognition by the immune system. Analysis of the normalized expression data from the 479 samples in TCGA melanoma cohort (RNA-seq) showed a clear positive correlation between the expression of MITF and ADAM10 with a Spearman coefficient of 0.34 (*p* = 1.44e^− 14^) and a Pearson coefficient of 0.37 (*p* = 6.57e^− 17^) (Fig. S5b). This degree of positive correlation is similar to that between expression of *MITF* and its classical target genes such as *TYR* and *MLANA*. Consistent with this, RT-PCR analysis of a panel of several melanoma cell lines showed a clear relationship between MITF and ADAM10 mRNA expression (Fig. S5c). Cell lines expressing higher levels of MITF (501mel, G631, IGR37, SK-MEL-28) also expressed more ADAM10, whereas SK-MEL-2, A375, and IGR39 cells with very low expression of MITF were found to have the lowest expression of ADAM10, with expression highly correlated (Fig S5c). The relationship between ADAM10 and MITF mRNA levels was recapitulated at the protein level (Fig. 5d). Of note, the IGR37 and IGR39 cell lines are derived from the same patient, with the non-invasive MITF^High^ IGR37 line being isolated from a metastatic lymph node whereas the invasive MITF^Low^ IGR39 line originates from the primary tumor. Consistent with this, the IGR39 cells also express high levels of N-cadherin, a hallmark of an invasive phenotype in melanoma (Fig. 5e). To gain further insight in the regulation of ADAM10 by MITF, we established human IGR37 melanoma cells in which *MITF* was knocked out using CRISPR/Cas9 (Fig. S3b). Compared to the parental cell line, in three different knockout clones low MITF substantially increased N-cadherin expression, but also strongly down-regulated ADAM10 (Fig. 5f). Similarly, silencing of MITF in SK-MEL-28 cells using two different siRNAs also resulted in a significant reduction of ADAM10 protein (Fig 5g).

### MITF-driven phenotypic plasticity modulates melanoma NK cell-mediated killing

**Fig. 6:**
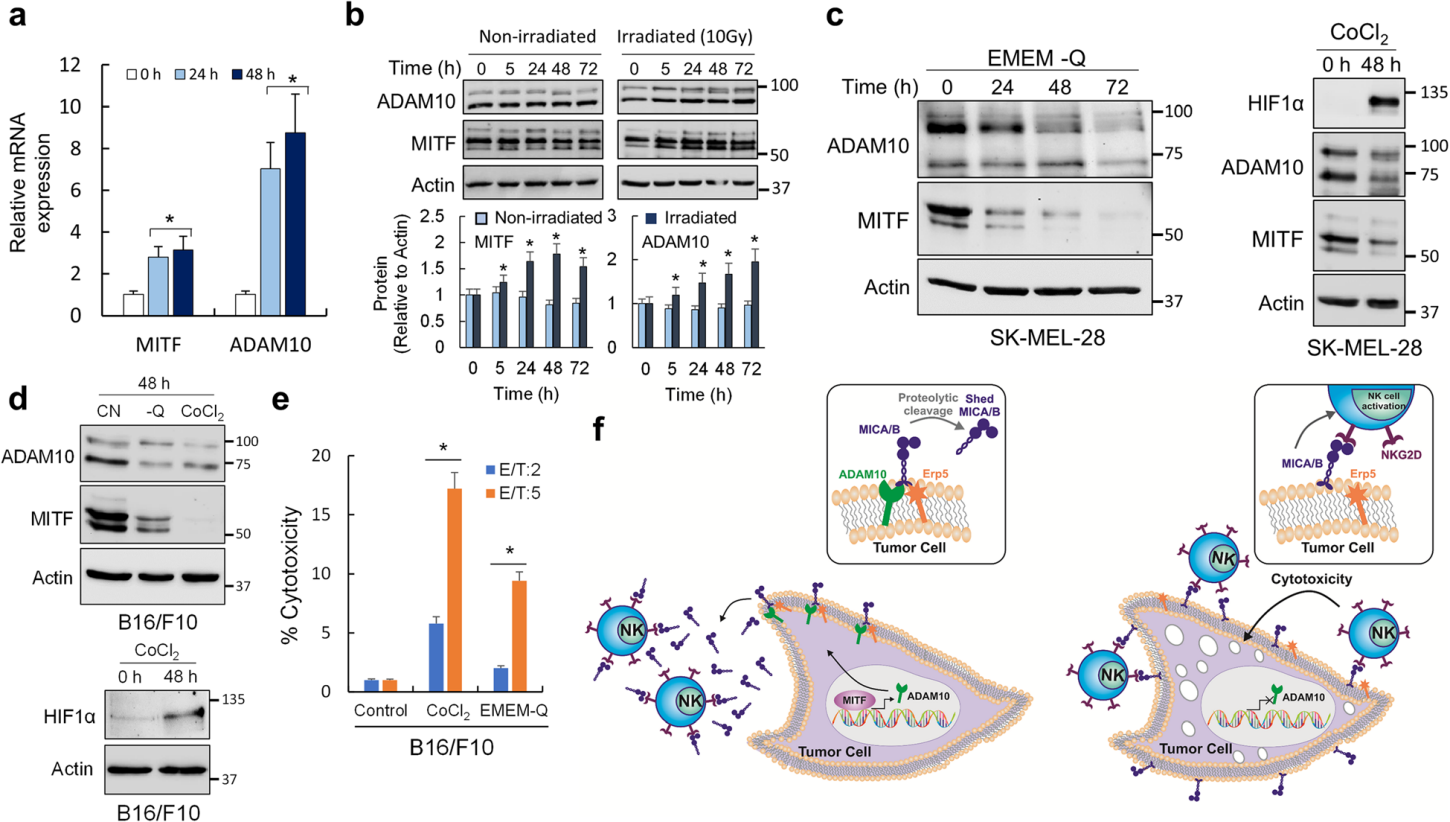
MITF-driven phenotypic plasticity modulates ADAM10 expression in melanoma. **a** Quantification of MITF and ADAM10 mRNA in SK-MEL-28 after 24 and 48 h of irradiation (10 Gy). Differences in MITF and ADAM10 mRNA expression at 24 and 48 h were statistically significant (*p < 0.05) with respect to the non-irradiated control (0 h). **b** Analysis of MITF and ADAM10 proteins in SK-MEL-28 cells after irradiation at different post-IR times. The histograms represent the relative amount of proteins with respect to actin. The IR-dependent increase in MITF and ADAM10 was only statistically significant in irradiated cells (*p < 0.05 at all assayed times with respect to non-irradiated controls). **c, d** Depletion of glutamine or CoCl_2_ treatments, to mimic hypoxia conditions, resulted in decreased levels of MITF and ADAM10 in human and mouse melanoma cells. **e** Depletion of glutamine and hypoxia sensitizes B16/F10 melanoma cells to NK-driven cytotoxicity. Spleens of untreated C57BL/6 were used for NK purification. Purified NKs were activated with IL-2 during 2 days. Then, IL-2-activated NKs were co-cultured with irradiated melanoma cells previously subjected to depletion of glutamine or CoCl_2_ (48 h treatments). NK cytotoxicity was evaluated by a ^51^Cr-release assay at two effector/target ratios (E/T). The results are representative of three independent experiments (*p < 0.05). **f** Schematic illustrating proposed mechanism by which MITF induces escape from innate immunity in melanoma. MITF expression in melanoma (left panels) directly upregulates ADAM10. MICA/B interacts with Erp5 and is shed by ADAM10 from the cell surface, which subsequently leads to a blocking of NKG2D. Lack of MITF expression in melanoma (right panels) downregulates ADAM10 and facilitates NKG2D-mediate immune response. Blots in this figure were cropped from different gels. Full blots are shown in Fig. S9 and quantified in Fig. S8

The results so far suggested that MITF would modulate the innate NK cell-mediated immune response via the ability of MITF to induce ADAM10 expression. Since irradiation induces MITF expression, and ADAM10 is an MITF target gene we would expect ADAM10 levels to increase following irradiation. To test this, SK-MEL-28 cells were subjected to a dose of 10 Gy and the levels of MITF and ADAM10 mRNA were analyzed at different time post-IR by qRT-PCR (Fig. 6a) and protein levels by Western blotting (Fig. 6b). The results confirmed that ADAM10 mRNA and protein levels were increased by irradiation. These results could in part explain the increased proteolytic cleavage of NKG2D ligands in irradiated melanoma cells (Figs. 4a-c) and the highly susceptibility of B16/F10-MITF-KO cells to NK-mediated cytotoxicity (Fig. 4d).

Tumor expansion coupled with a poorly organized tumor vasculature often results in areas of limited nutrient supply and hypoxia and variations in the nutritional microenvironment contribute to tumor heterogeneity and therapeutic response [2]. Many microenvironmental stresses can suppress MITF expression including hypoxia, inflammatory signaling, and reduced glucose or glutamine availability [15, 17, 18]. We therefore asked whether decreased MITF expression mediated by CoCl_2,_ to mimic hypoxia, or glutamine limitation could suppress ADAM10 expression. We observed that reduced glutamine in SK-MEL-28 cells reduced both MITF and ADAM10 expression (Fig. 6c, left panel). CoCl_2_ increased HIF1α expression and decreased MITF and ADAM10 (Fig. 6c, right panel). Similar results were also observed in B16/F10 cells (Fig. 6d). Consistent with the effects of these microenvironmental triggers on ADAM10 expression, both CoCl_2_ and glutamine limitation strongly enhanced NK cell-mediated killing of irradiated B16/F10 cells (Fig. 6e).

### Targeting ADAM10 as a rational anti-melanoma immunotherapy

**Fig. 7:**
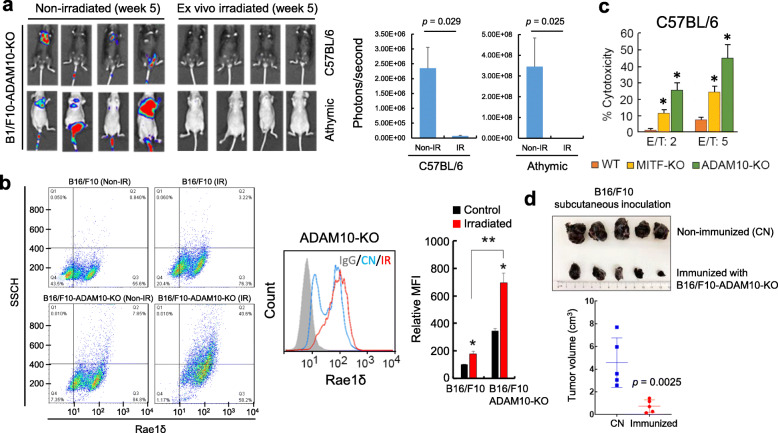
Knock out of ADAM10 and its effects on mice immunity. **a** Luciferase imaging of C57BL/6 and athymic mice injected in the tail vein with B16/F10-ADAM10-KO cells. When indicated, cells were irradiated ex vivo (10 Gy; 24 h) before injection. Color scale was set to Min = 1.00e6; Max = 1.00e7 for all pictures. In histograms *p*-values were calculated by Mann-Whitney tests. **b** Flow cytometry determination of Rae1δ in B16/F10 and effects of ADAM10 depletion. Cells were non-irradiated or irradiated (IR, 10 Gy, 24 h). Histogram represents MFI (Mean Fluorescence Intensity) in assayed conditions. *p < 0.05 when compared with their respective non-irradiated cells. **p < 0.005 when comparing both irradiated cells. **c** Depletion of ADAM10 sensitizes B16/F10 melanoma cells to NK-driven cytotoxicity. Spleens of untreated C57BL/6 were used for NK purification as indicated in Fig. 6e. IL-2-activated NKs were co-cultured with indicated melanoma cells. To promote MITF activity, target cells B16/F10 (WT) and B16/F10-ADAM10-KO (ADAM10-KO) were previously irradiated (10 Gy, 24 h). The results of NK cytotoxicity on irradiated B16/F10-MITF-KO (MITF-KO) are included for comparison. NK cytotoxicity was evaluated by a ^51^Cr-release assay. The results are representative of three independent experiments (*p < 0.05). **d** Vaccination assay. Mice were non-immunized (CN group) or immunized with ex vivo irradiated (10 Gy; 24 h before injection) B16/F10-ADAM10-KO (immunized group). Then, non-irradiated B16/F10 melanoma cells were subcutaneously injected and the volume of tumors analyzed after 4 weeks. The histogram represents the tumor volume in control and immunized groups (*n* = 5). Grouped data passed the Shapiro-Wilk normality test

Our results suggest that MITF can be considered as a double-edged sword in the anti-melanoma immune response. Although MITF expression may result in increased recognition of melanoma cells by the adaptive system through the activation and presentation of specific antigens (such as MLANA), MITF expression may also facilitate immune evasion by activating ADAM10 expression to prevent NKG2D receptor activation in NK cells (Fig. 6f). In this context, inhibiting ADAM10 activity, but maintaining MITF transcriptional function could represents a rational strategy to guide melanoma cells to their destruction by the immune system. To explore this hypothesis we used CRISPR-Cas9 technology to generate several ADAM10-knock-out B16/F10 cell lines and confirmed that ADAM10-KO cells maintained MITF expression (Fig. S6). First, we observed that a B16/F10-ADAM10-KO cell line (clone F4; Fig. S6) established consistent tumors in both C57BL/6 and athymic mice when was injected in the tail vein, but not when the cells were irradiated ex vivo before injection (Fig. 7a). Unlike irradiated parental B16/F10 melanoma cells which form lung tumors in nude mice but not in immunocompetent mice owing to T-cell-mediated immune rejection (Fig. 1), the ADAM10-KO cells behave like MITF-KO cells in that they fail to form tumors in nude mice. Consistent with the irradiated B16/F10-ADAM10-KO cell line being rejected by NK-mediated cell killing, the ADAM10-KO cells expressed elevated levels of the NKG2D ligand Rae1δ to even higher levels than cells lacking MITF (B16/F10-MITF-KO) (Fig. 7b). Moreover, an in vitro cytotoxicity assay revealed that IL-2 activated NK cells purified from C57BL/6 mouse spleens exhibited enhanced killing of B16/F10-ADAM10-KO cells compared to B16/F10-MITF-KO cells, which was in turn higher than that achieved against parental B16/F10 cells (Fig. 7c). This is consistent with the MITF-KO incompletely suppressing ADAM10 expression, whereas the ADAM10-KO would prevent all expression. However, while elevated susceptibility to NK-mediated killing was a characteristic of both MITF-KO and ADAM10-KO melanoma cells, a major difference was observed when we performed a vaccination assay. For this assay, untreated B16/F10 cells were subcutaneously injected into animals immunized 4 weeks before with irradiated B16/F10-ADAM10-KO cells (Fig. 7d). Compared with control mice, the results showed an evident immune response against B16/F10 cells with lower growth of tumours in the group of mice immunized with irradiated B16/F10-ADAM10-KO cells. This experiment is important since B16/F10-ADAM10-KO cells maintain high levels of MITF. Since ADAM10-KO cells exhibit high levels of NKG2D ligands, they will be efficiently targeted by NK-mediated cell killing, while maintenance of MITF expression will facilitate a T-cell-mediated anti-tumor response. Taken together, the results indicate that MITF expression can promote the evasion of NK cell-driven tumor immunity through the proteolytic activity of ADAM10 on NKG2D ligands.

## Discussion

Cutaneous melanoma is widely considered one of the most immunogenic types of cancer based on its high mutation frequency, its frequent spontaneous remission at an early stage, and lymphocytic infiltrations into primary tumors and metastases that correlates with better outcome owing to T-cell recognition of melanoma antigens [42]. As a consequence, immunotherapy based on blocking co-inhibitory receptors such as CTLA-4 and PD-1 expressed on the T-lymphocyte surface has emerged as a powerful strategy in melanoma treatment with the development of several checkpoint blocking antibodies such as Ipilimumab or Pembrolizumab. However, despite the success of T-cell-based immunotherapies, a significant proportion of patients either do not respond or resistance emerges leading to disease progression [3]. Although many factors, including deficient antigen processing and presentation, may increase resistance to immunotherapies, a key contributing factor is melanoma phenotypic plasticity that enables cells to survive by adapting their gene expression programs when confronted with a hostile microenvironment. Significantly, for immunotherapies that rely on recognition of melanoma antigens, a range of microenvironmental stresses, including inflammation, hypoxia and nutrient limitation can lead to down-regulation of MITF, melanoma dedifferentiation, loss of melanoma antigen expression and the emergence of immunotherapy resistance [43]. Given that MITF controls many aspects of melanoma biology [22], including expression of differentiation-associated antigens such as MLANA [31], microenvironmental stresses that lead to low MITF expression are associated with resistance to conventional T-cell based immunotherapies, despite their frequently high levels of T-cell infiltration [11]. In particular, inflammatory signaling arising from activated infiltrating immune cells can lead to MITF down-regulation and consequently immune escape [17, 20]. There is therefore an urgent need for complementary approaches to activate an anti-tumor response, especially for those that take into account melanoma phenotypic heterogeneity.

Our results have identified a key role for MITF in controlling NK-mediated cell death by directly regulating the expression of the ADAM10 protease. Since ADAM10 suppresses the presentation of the NKG2D ligands for NK cells on the melanoma cell surface, loss of either MITF or ADAM10 increased ligand expression and consequently greatly increased NK cell-mediated cell death. These observations raise the possibility of using small molecules to target the ADAM10 protease to enhance NK cell-mediated melanoma killing. This approach would have an advantage in that MITF expression is maintained, enabling continued expression of MITF target genes encoding immunogenic differentiation-associated melanoma antigens such as MLANA. Indeed, harnessing NK cells for anti-cancer immunotherapy has a number of key advantages, not least that one of their fundamental functions is to target cells lacking expression of MHC class I molecules [44] which would be refractory to T-cell-mediated killing. Targeting ADAM10 would therefore represent a complementary approach to current T-cell based immunotherapies.

The identification of ADAM10 as an MITF target gene, suggests that MITF may play a dual role in shaping the anti-melanoma immune response, both promoting expression of antigens like MLANA known to elicit a robust T-cell response, but at the same time suppressing NK-cell-mediated killing. Since suppression of MITF activity is linked to invasiveness and tumor-initiating capacity in melanoma [45], presumably invasive or circulating MITF^Low^ cells would be more susceptible to NK-mediated cell death than proliferating or more differentiated MITF^High^ cells. However, the role of MITF in suppressing NK cell-mediated cell death likely reflects its physiological role in skin melanocytes. Exposure of the skin to UV irradiation drives up-regulation of MITF via keratinocyte-mediated expression of melanocyte-stimulating hormone and downstream cAMP signaling [46] to promote expression of genes associated with pigmentation and melanosome transfer from melanocytes to keratinocytes where they protect against DNA damage arising from subsequent irradiation [47]. Given the ability of NK cells to target skin melanocytes expressing a tyrosinase hapten [48], it seems plausible that the ability of MITF to promote ADAM10 expression represents a means to suppress NK cell-mediated killing of differentiated melanocytes following solar UV irradiation and thereby maintain the integrity of the protective tanning response.

In addition to its roles in suppressing NK cell-mediated killing and expression of melanoma antigens, MITF has an additional role in regulating the immune response by suppressing the expression of an inflammatory secretome [49] that can lead to effects on immune cell attraction [50]. This can arise in large part via MITF-mediated regulation of the *SCD* gene that promotes long fatty acid desaturation to maintain the correct ratio of membrane saturated and mono-unsaturated fatty acids [51]. Low expression of SCD, for example caused by a reduction in MITF levels, can drive expression of an immune modulatory and pro-inflammatory secretome mediated by the stress activated transcription factors ATF4 and NFκB [51]. The central role of MITF in melanoma biology as well as in shaping the immune response means that approaches combining inhibition of ADAM10 with manipulation of MITF levels, for example to sensitize cells to therapy [23], may provide an avenue towards more effective immune therapy.

## Conclusion

In summary, our results provide a novel insight into how melanoma cells engage in bidirectional interactions with the immune system, highlight the importance of MITF and melanoma phenotype in shaping the anti-melanoma immune response, and suggest that targeting ADAM10 may represent a complementary therapeutic approach to current T-cell-based immunotherapies.

## Supplementary Information

**Additional file 1:** **Figure S1.** Depletion of CD8+ T lymphocytes. C57BL/6 mice were treated intraperitoneally with a dose (250 μg) of IgG isotype control b or anti-CD8 (clone 2.43) a, c monoclonal antibodies every 3 days. After four doses, peripheral blood was collected and stained to test the presence of CD8+ cytotoxic T lymphocytes among lymphoid cells (R^− 1^) by flow cytometry. Data shows a complete clearance of T CD8+ cells in a representative experiment out of two with similar results. The remaining blood cell counts (granulocyte, monocytes, NK and B lymphocytes) were unchanged (data not shown).

**Additional file 2:** **Figure S2.** IR modulates the immune responsiveness of melanoma cells. a Effects of IR (10 Gy) on HLA-I proteins assayed by confocal microscopy (the scale bar, 15 μm, refers to both panels). b Effects of IR on HLA-I proteins assayed by flow cytometry (IR, 10 Gy, 48 h). c Effects of IR on HLA-I proteins assayed by qRT-PCR (IR, 10 Gy); *p < 0.05 when compared with its respective control (ns, no significant). d Confocal microscopy analysis of calreticulin (CALR) and MLANA before and after IR (IR, 24 h). The scale bar (15 μm), refers to both panels.

**Additional file 3: Figure S3.** *MITF* gene knockout using the CRISPR/Cas9 system in B16/F10-luc2 and IGR37 cells. **a**, **b** The schematic diagrams show the guide RNA (gRNA) targeting site on exon 3 of the *Mitf* mouse gene and exon 2 of *MITF* human gene. Protospacer adjacent motif (PAM) sequences are also presented. The figures also show Sanger sequencing analysis of PCR fragments amplified from gRNA target regions (the inserted nucleotide is in blue) and protein sequence in wild type (WT) and knockout (KO) cells. **c** Protein expression in WT and selected clones was assayed by western blot. Full blots are shown in Fig. S9. Histograms represent protein quantification. **d** Morphological aspect of WT and knockout melanoma cells used in this study. Bars, 100 μm. **e** Functional characterization (proliferation and apoptosis) of knockout cells compared to their respective WT cells. MITF knockout in melanoma cells did not induce significant apoptosis (ns, no significant). Treatment of indicated cells with doxorubicin (1 μM; 24 h) was included as an apoptosis positive control; *p < 0.05 when compared with their respective untreated WT cells. **f** Effect of IR on MITF expression in B16/F10 and B16/F10-MITF-KO. Histogram represents protein quantification. Full blots are shown in Fig. S9.

**Additional file 4:** **Figure S4.** B16/F10-MITF-KO cells were able to form tumors in different experimental models. a Luciferase imaging of R2G2 mice injected in the tail vein with B16/F10 or B16/F10-MITF-KO cells. Picture are representative of *n* = 3 per group. Color scale was set to Min = 1.00e6; Max = 1.00e7 for all pictures. b Luciferase imaging after subcutaneous injection of indicated melanoma cells induced tumors in C57BL/6 mice. Picture are representative of n = 3 per group. **c** Luciferase imaging of athymic mice injected in the tail vein with B16/F10-luc2-puromycin cells. Cells were irradiated ex vivo (10 Gy; 24 h). (*n* = 4).

**Additional file 5:** **Figure S5.** Relationship between MITF and ADAM10 in different melanoma cell lines. a The ChIP-Seq data in 501mel cells [40] indicate the sequence of binding of MITF (E-Box) to the ADAM10 promoter. b The analysis of the normalized expression data (RNA-Seq) shows a positive correlation for the expression of MITF and ADAM10 in melanoma. **c** Relationship of MITF and ADAM10 mRNAs in different melanoma cell lines. The mRNA profiles have been calculated in relation to the actin of each sample.

**Additional file 6:** **Figure S6.** *Adam10* gene knockout using the CRISPR/Cas9 system in B16/F10 cells. The schematic diagram shows the guide RNA (gRNA) targeting site on exon 3 for clone F4 and exon 2 for clone B6 of the mouse *Adam10* gene. Protospacer adjacent motif (PAM) sequences are also presented. The figure also shows Sanger sequencing analysis of PCR fragments amplified from gRNA target regions (the inserted nucleotide is in blue) and protein sequence in wild type (WT) and knockout (KO) cells. Protein expression in WT and two selected clones (F4 and B6) was assayed by western blot. Full blots are shown in Fig. S9. Histogram represents protein quantification.

**Additional file 7:** **Figure S7.** Quantification of both MITF and MLANA in flow cytometry experiments depicted in Fig. 2a. *p < 0.05 when compared with their respective untreated controls.

**Additional file 8:** **Figure S8.** Densitometry analysis of Western blot data presented in the indicated figures.

**Additional file 9:** **Figure S9.** The figure shows the full original and uncropped images for the western blots presented in the indicated figures. The identification of corresponding protein bands was based on the expected molecular weight as indicated in the main figure.

## Data Availability

All data generated or analyzed during this study are included in this published article (and its supplementary information files).

## Abbreviations

APC: Allophycocyanin
ADAM10: A Disintegrin and metalloproteinase domain-containing protein 10
ATF4: Activating transcription factor 4
cAMP: Cyclic adenosine monophosphate
Cas9: CRISPR-associated protein 9
ChIP: Chromatin immunoprecipitation
ChIP-seq: Chromatin immunoprecipitation – sequencing
CRISPR: Clustered regularly interspaced short palindromic repeats
CTLA-4: Cytotoxic T-lymphocyte antigen 4
EMEM: Eagle’s minimum essential medium
ELISA: Enzyme-linked immunosorbent assay
FBS: Fetal bovine serum
GAPDH: Glyceraldehyde-3-phosphate dehydrogenase
gRNA: Guide RNA
HDAC3: Histone Deacetylase 3
HDR: Homology directed repair
H&E: hematoxylin and eosin
HIF1α: Hypoxia inducible factor 1α
IHC: Immunohistochemistry
IL-2: Interleukin-2
IR: Irradiation
KO: Knock-out
Luc: Luciferase
MICA: MHC class I polypeptide-related sequence A
MICB: MHC class I polypeptide-related sequence B
MHC: Major histocompatibility complex
MITF: Melanocyte Inducing Transcription Factor
NFκB: Nuclear factor-kappa B
NK: Natural killer
NKG2D: Natural killer group 2, member D receptor
PAM: Protospacer adjacent motif
PBS: Phosphate-buffered saline
PD-1: Programmed death–1
qPCR: Quantitative PCR
R2G2: Radioresistant Rag2-IL2rg
RT-PCR: Real time-PCR
RT-qPCR: Real time quantitative PCR
SCD: Stearoyl-CoA desaturase
SDS-PAGE: Sodium dodecyl sulfate polyacrylamide gel electrophoresis
siRNAs: Small interfering RNAs
TCGA: The cancer genome atlas
TYR: Tyrosinase
UV: Ultraviolet
WT: Wild type
