# Supplementary material for: MITF induces escape from innate immunity in melanoma
Source: J Exp Clin Cancer Res. 2021 Mar 31;40:117. doi: 10.1186/s13046-021-01916-8 (PMC8015040; doi:10.1186/s13046-021-01916-8)

Figure S1. Depletion of CD8+ T lymphocytes. C57BL/6 mice were treated intraperitoneally with a dose (250 µg) of IgG isotype control **b** or anti-CD8 (clone 2.43) **a, c** monoclonal antibodies every 3 days. After four doses, peripheral blood was collected and stained to test the presence of CD8+ cytotoxic T lymphocytes among lymphoid cells (R-1) by flow cytometry. Data shows a complete clearance of T CD8+ cells in a representative experiment out of two with similar results. The remaining blood cell counts (granulocyte, monocytes, NK and B lymphocytes) were unchanged (data not shown).

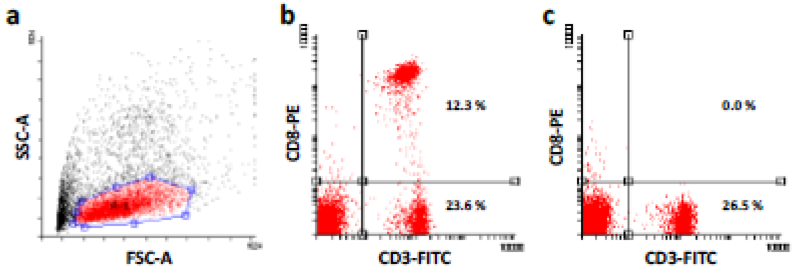

Supplement: Supplementary file 1 [file 13046_2021_1916_MOESM1_ESM.docx]
