# Supplementary material for: MITF induces escape from innate immunity in melanoma
Source: J Exp Clin Cancer Res. 2021 Mar 31;40:117. doi: 10.1186/s13046-021-01916-8 (PMC8015040; doi:10.1186/s13046-021-01916-8)

Figure S2. IR modulates the immune responsiveness of melanoma cells. **a** Effects of IR (10 Gy) on HLA-I proteins assayed by confocal microscopy (the scale bar, 15 µm, refers to both panels). **b** Effects of IR on HLA-I proteins assayed by flow cytometry (IR, 10 Gy, 48 h). **c** Effects of IR on HLA-I proteins assayed by qRT-PCR (IR, 10 Gy); *$p < 0.05$ when compared with its respective control (ns, no significant). **d** Confocal microscopy analysis of calreticulin (CALR) and MLANA before and after IR (IR, 24 h). The scale bar (15 µm), refers to both panels.

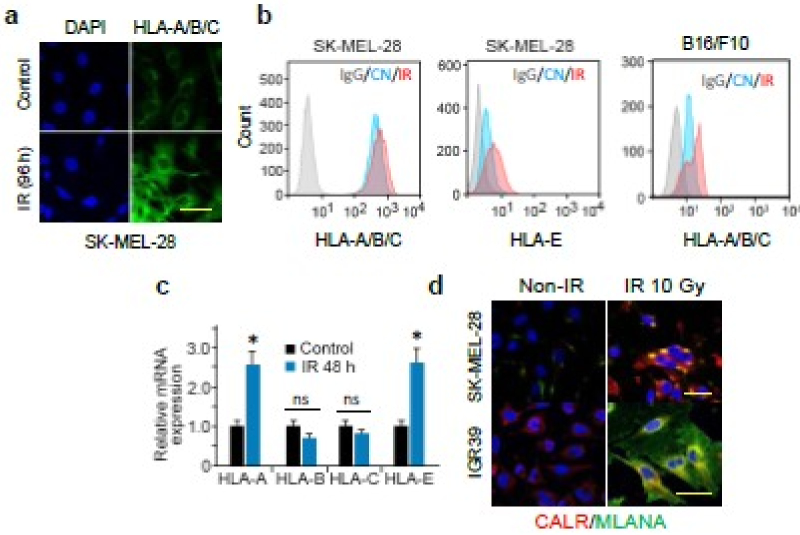

Supplement: Supplementary file 2 [file 13046_2021_1916_MOESM2_ESM.docx]
