# Supplementary material for: MITF induces escape from innate immunity in melanoma
Source: J Exp Clin Cancer Res. 2021 Mar 31;40:117. doi: 10.1186/s13046-021-01916-8 (PMC8015040; doi:10.1186/s13046-021-01916-8)
Supplement: Supplementary file 3 [file 13046_2021_1916_MOESM3_ESM.docx]

Figure S3. *MITF* gene knockout using the CRISPR/Cas9 system in B16/F10-luc2 and IGR37 cells. **a, b** The schematic diagrams show the guide RNA (gRNA) targeting site on exon 3 of the *Mitf* mouse gene and exon 2 of *MITF* human gene. Protospacer adjacent motif (PAM) sequences are also presented. The figures also show Sanger sequencing analysis of PCR fragments amplified from gRNA target regions (the inserted nucleotide is in blue) and protein sequence in wild type (WT) and knockout (KO) cells. **c** Protein expression in WT and selected clones was assayed by western blot. Full blots are shown in Fig. S9. Histograms represent protein quantification. **d** Morphological aspect of WT and knockout melanoma cells used in this study. Bars, 100 µm. **e** Functional characterization (proliferation and apoptosis) of knockout cells compared to their respective WT cells. MITF knockout in melanoma cells did not induce significant apoptosis (ns, no significant). Treatment of indicated cells with doxorubicin (1 µM; 24 h) was included as an apoptosis positive control; $*p < 0.05$ when compared with their respective untreated WT cells. **f** Effect of IR on MITF expression in B16/F10 and B16/F10-MITF-KO. Histogram represents protein quantification. Full blots are shown in Fig. S9.

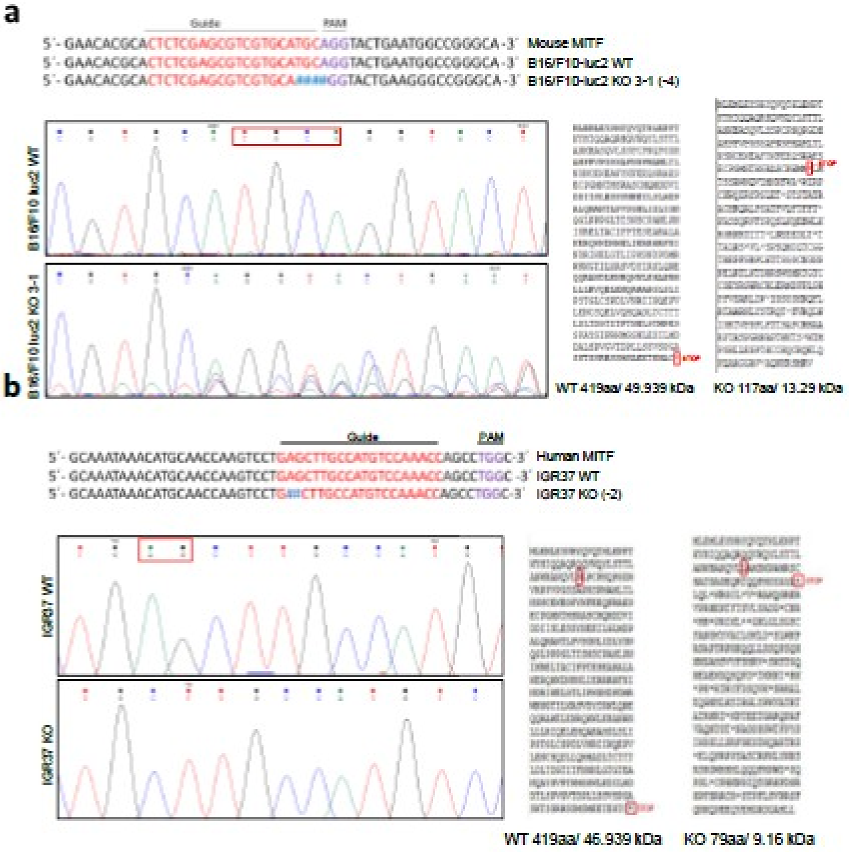

c

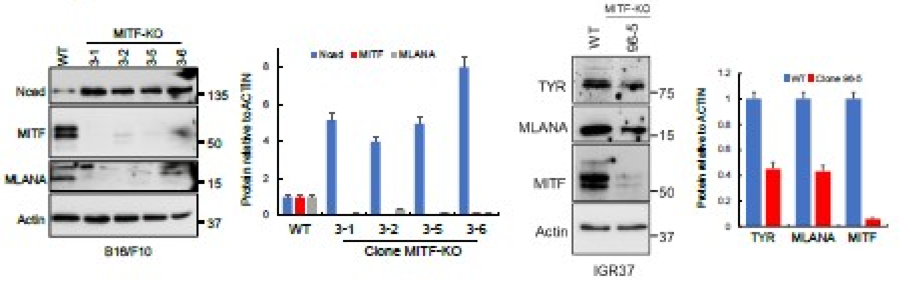

MITF-KO
WT
3-1
3-2
3-5
3-6
Ncad
MITF
MLANA
Actin
135
50
15
37
B16/F10
Ncad
MITF
MLANA
Protein relative to ACTIN
WT
Clone MITF-KO
TYR
IGR37
WT
Clone 96-5

d

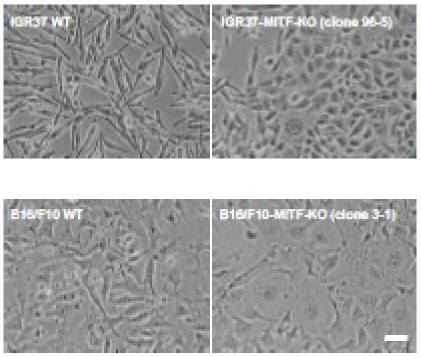

IGR37 WT
IGR37-MITF-KO (clone 96-5)
B16/F10 WT
B16/F10-MITF-KO (clone 3-1)

e

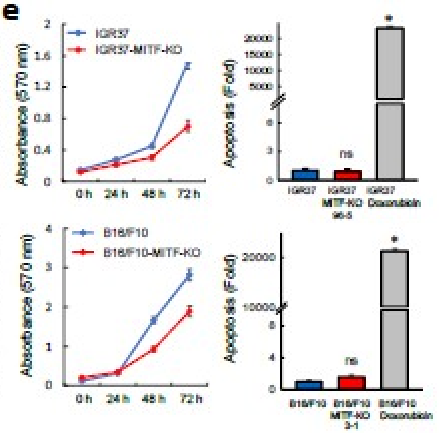

IGR37
IGR37-MITF-KO
Absorbance (570 nm)
0 h
24 h
48 h
72 h
Apoptosis (Fold)
ns
IGR37 Doxorubicin
B16/F10
B16/F10-MITF-KO
B16/F10 Doxorubicin

f

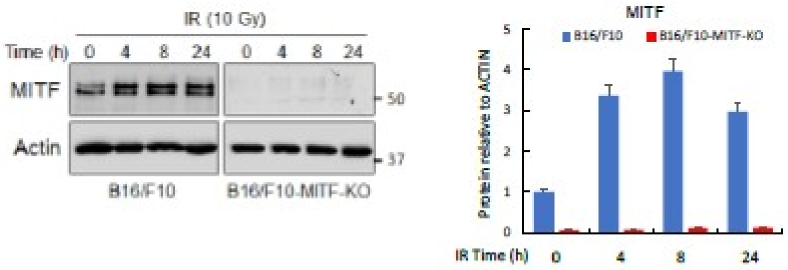

IR (10 Gy)
Time (h)
0
4
8
24
MITF
Actin
50
37
B16/F10
B16/F10-MITF-KO
MITF
Protein relative to ACTIN
IR Time (h)
