# Supplementary material for: MITF induces escape from innate immunity in melanoma
Source: J Exp Clin Cancer Res. 2021 Mar 31;40:117. doi: 10.1186/s13046-021-01916-8 (PMC8015040; doi:10.1186/s13046-021-01916-8)

Figure S4. B16/F10-MITF-KO cells were able to form tumors in different experimental models. **a** Luciferase imaging of R2G2 mice injected in the tail vein with B16/F10 or B16/F10-MITF-KO cells. Picture are representative of n = 3 per group. Color scale was set to Min = 1.00e6; Max = 1.00e7 for all pictures. **b** Luciferase imaging after subcutaneous injection of indicated melanoma cells induced tumors in C57BL/6 mice. Picture are representative of n = 3 per group. **c** Luciferase imaging of athymic mice injected in the tail vein with B16/F10-luc2-puromycin cells. Cells were irradiated *ex vivo* (10 Gy; 24 h). (n = 4).

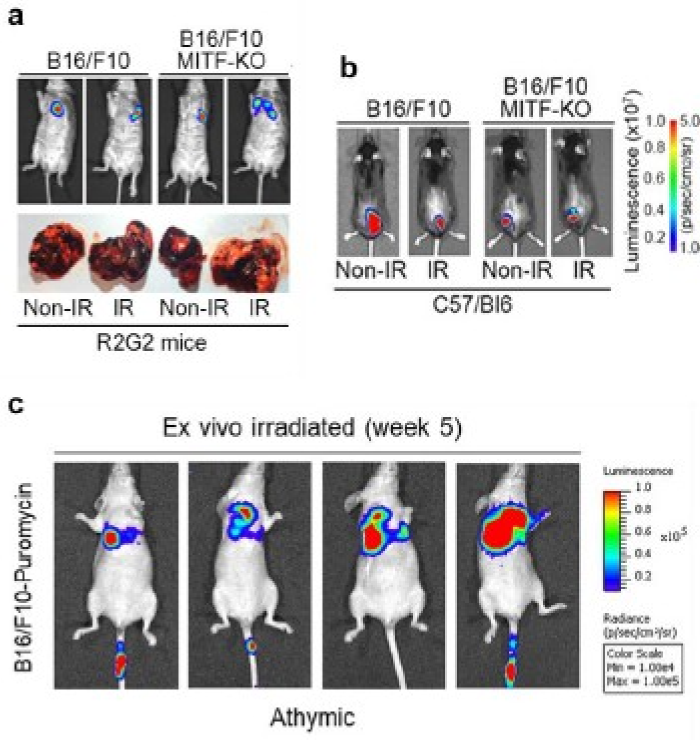

Supplement: Supplementary file 4 [file 13046_2021_1916_MOESM4_ESM.docx]
