# Supplementary material for: MITF induces escape from innate immunity in melanoma
Source: J Exp Clin Cancer Res. 2021 Mar 31;40:117. doi: 10.1186/s13046-021-01916-8 (PMC8015040; doi:10.1186/s13046-021-01916-8)

**Figure S5.** Relationship between MITF and ADAM10 in different melanoma cell lines. **a** The ChIP-Seq data in 501mel cells [40] indicate the sequence of binding of MITF (E-Box) to the ADAM10 promoter. **b** The analysis of the normalized expression data (RNA-Seq) shows a positive correlation for the expression of MITF and ADAM10 in melanoma. **c** Relationship of MITF and ADAM10 mRNAs in different melanoma cell lines. The mRNA profiles have been calculated in relation to the actin of each sample.

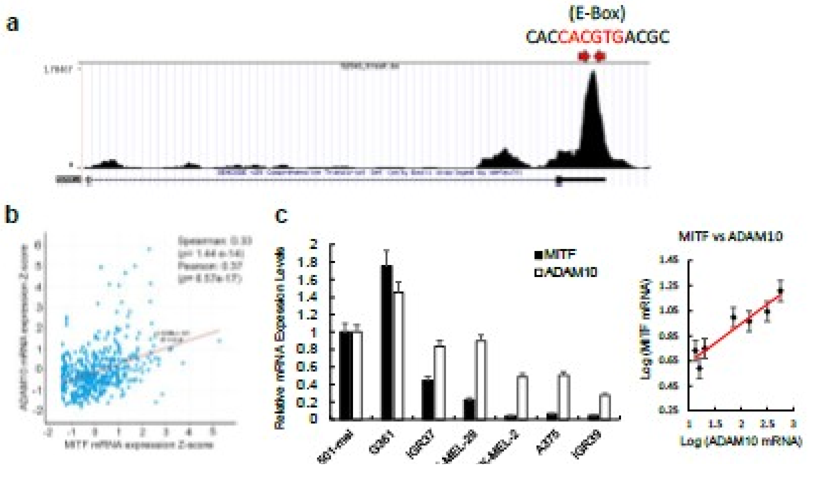

Supplement: Supplementary file 5 [file 13046_2021_1916_MOESM5_ESM.docx]
