# Supplementary material for: MITF induces escape from innate immunity in melanoma
Source: J Exp Clin Cancer Res. 2021 Mar 31;40:117. doi: 10.1186/s13046-021-01916-8 (PMC8015040; doi:10.1186/s13046-021-01916-8)

Figure S6. *Adam10* gene knockout using the CRISPR/Cas9 system in B16/F10 cells. The schematic diagram shows the guide RNA (gRNA) targeting site on exon 3 for clone F4 and exon 2 for clone B6 of the mouse *Adam10* gene. Protospacer adjacent motif (PAM) sequences are also presented. The figure also shows Sanger sequencing analysis of PCR fragments amplified from gRNA target regions (the inserted nucleotide is in blue) and protein sequence in wild type (WT) and knockout (KO) cells. Protein expression in WT and two selected clones (F4 and B6) was assayed by western blot. Full blots are shown in Fig. S9. Histogram represents protein quantification.

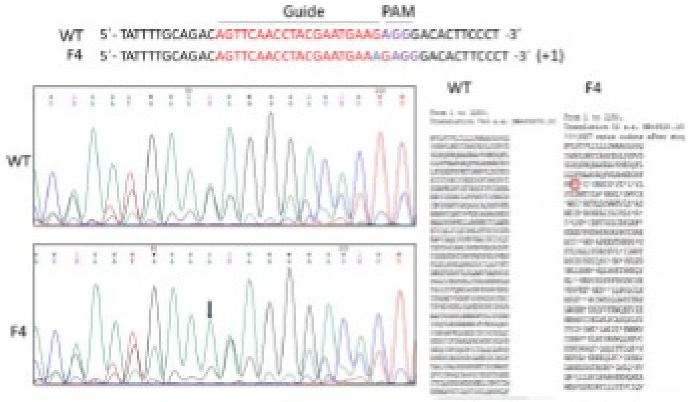

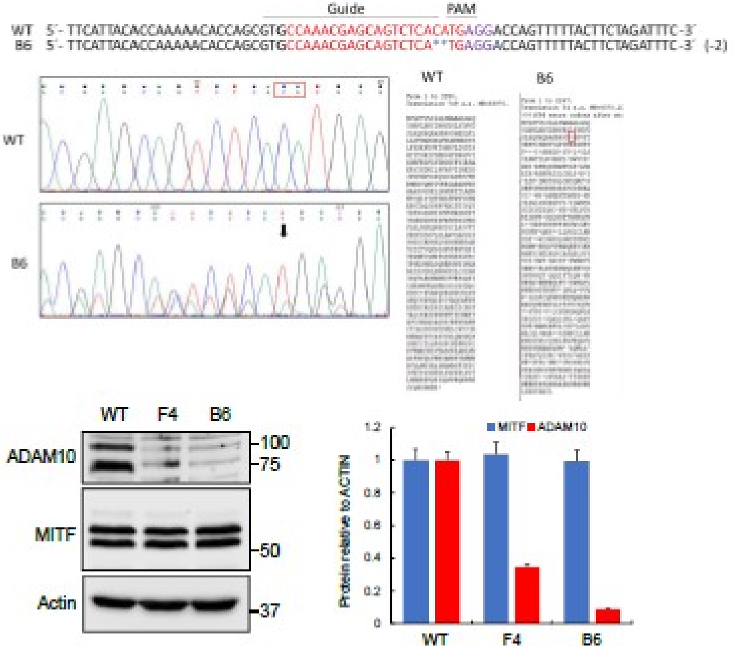

Supplement: Supplementary file 7 [file 13046_2021_1916_MOESM7_ESM.docx]
