# Supplementary material for: MITF induces escape from innate immunity in melanoma
Source: J Exp Clin Cancer Res. 2021 Mar 31;40:117. doi: 10.1186/s13046-021-01916-8 (PMC8015040; doi:10.1186/s13046-021-01916-8)

**Figure S8.** Densitometry analysis of Western blot data presented in the indicated figures.

Fig. 2b

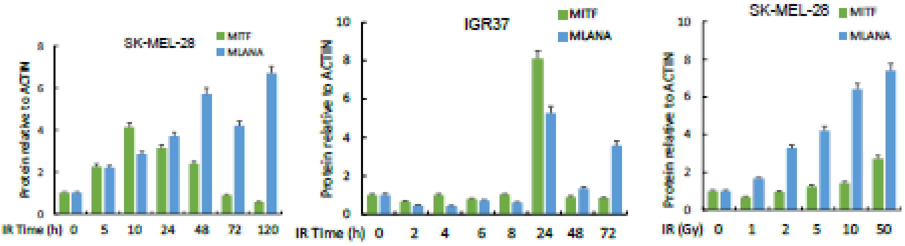


Fig. 2c

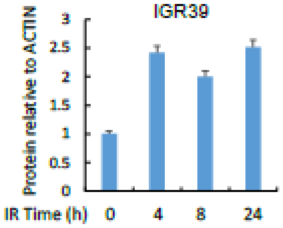


Fig. 5c

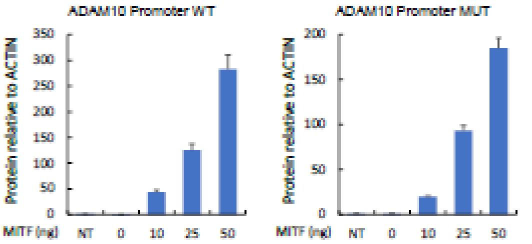


Fig. 5d

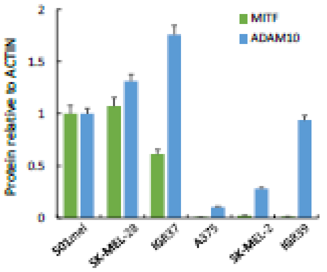


Fig. 5e

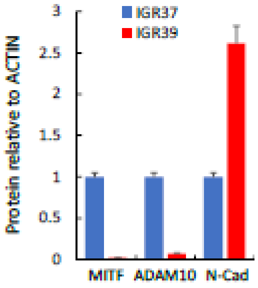


Fig. 5f

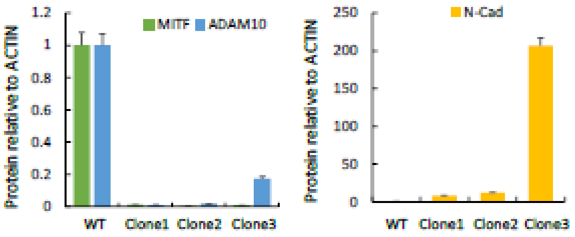

Fig. 5g

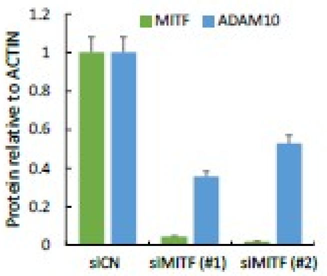


Fig. 6c

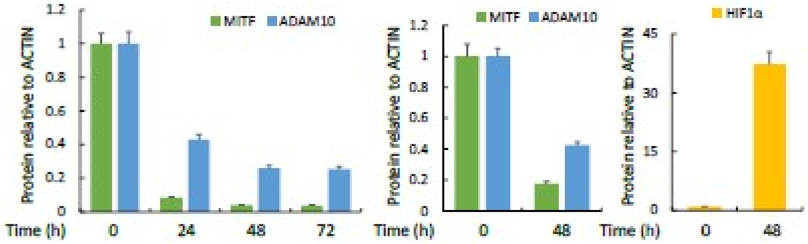


Fig. 6d

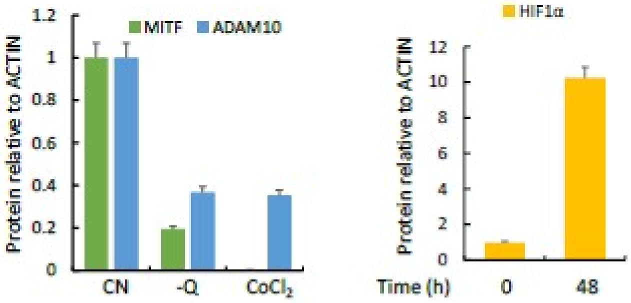

Supplement: Supplementary file 8 [file 13046_2021_1916_MOESM8_ESM.docx]
