# Supplementary material for: MITF induces escape from innate immunity in melanoma
Source: J Exp Clin Cancer Res. 2021 Mar 31;40:117. doi: 10.1186/s13046-021-01916-8 (PMC8015040; doi:10.1186/s13046-021-01916-8)

Figure S9. The figure shows the full original and uncropped images for the western blots presented in the indicated figures. The identification of corresponding protein bands was based on the expected molecular weight as indicated in the main figure.

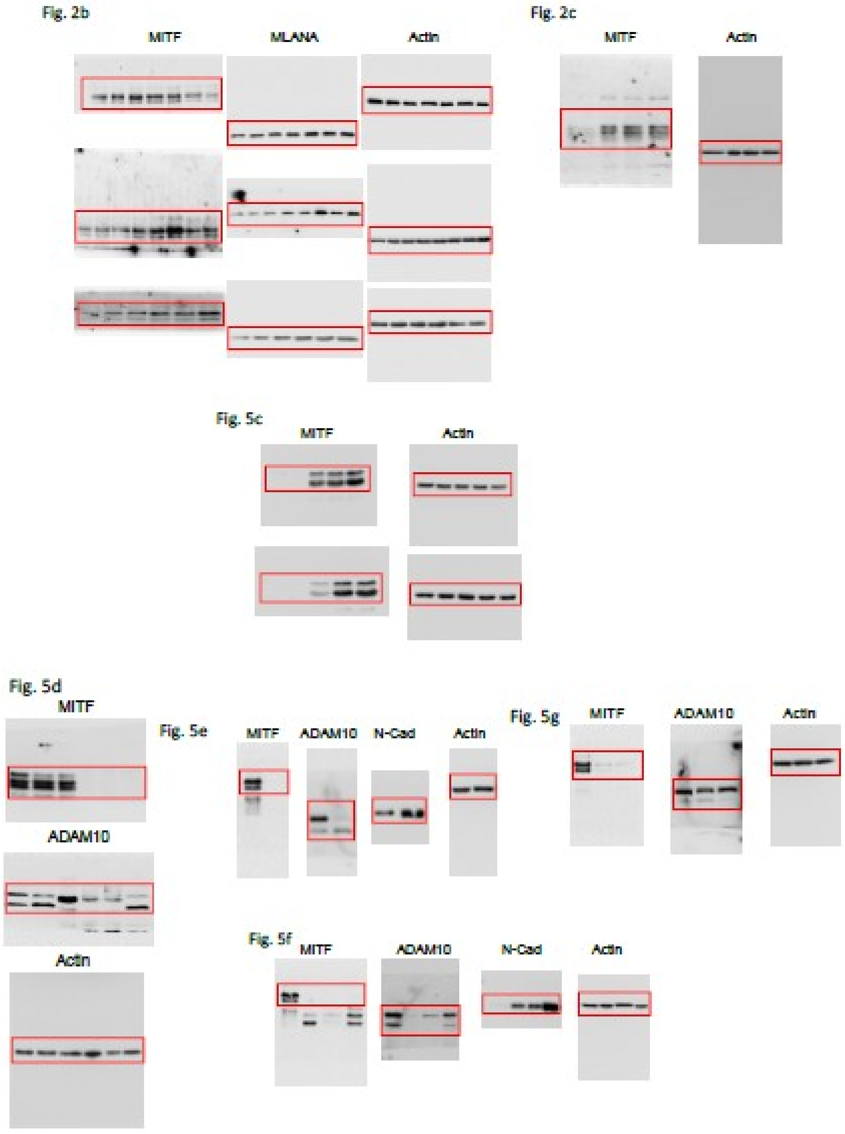

Fig. 6b

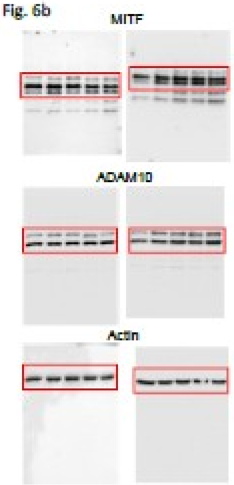

Fig. 6c

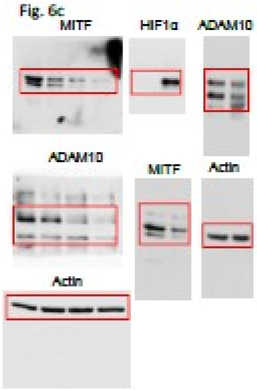

Fig. 6d

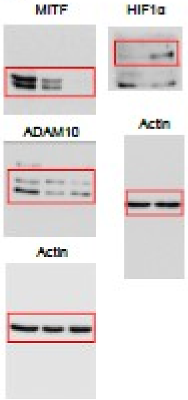

Fig. S3c

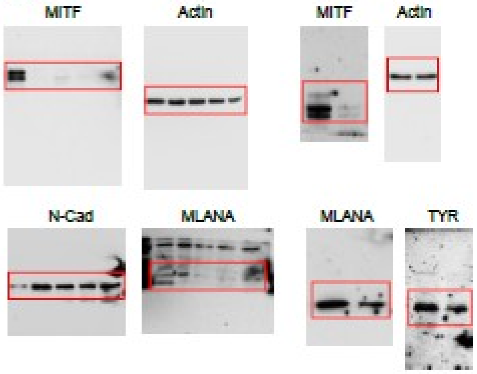

Fig. S3f

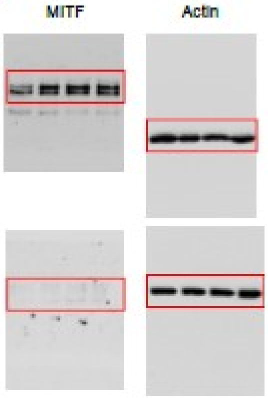

Fig. S6

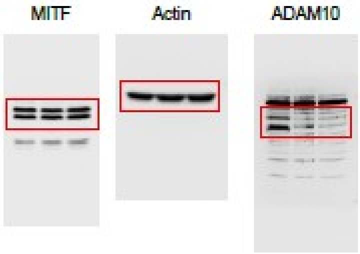

Supplement: Supplementary file 9 [file 13046_2021_1916_MOESM9_ESM.docx]
